# Recognize the Value of the Sum Score, Psychometrics’ Greatest Accomplishment

**DOI:** 10.1007/s11336-024-09964-7

**Published:** 2024-04-17

**Authors:** Klaas Sijtsma, Jules L. Ellis, Denny Borsboom

**Affiliations:** 1 Department of Methodology and Statistics TSB, Tilburg University, PO Box 90153, 5000LE Tilburg, The Netherlands; 2 Open University OF THE NETHERLANDS, Heerlen, The Netherlands; 3 University of Amsterdam, Amsterdam, The Netherlands

**Keywords:** classical test theory, factor analysis model, item response theory, latent variable, lower bound to reliability, network models, reliability, sum score

## Abstract

The sum score on a psychological test is, and should continue to be, a tool central in psychometric practice. This position runs counter to several psychometricians’ belief that the sum score represents a pre-scientific conception that must be abandoned from psychometrics in favor of latent variables. First, we reiterate that the sum score stochastically orders the latent variable in a wide variety of much-used item response models. In fact, item response theory provides a mathematically based justification for the ordinal use of the sum score. Second, because discussions about the sum score often involve its reliability and estimation methods as well, we show that, based on very general assumptions, classical test theory provides a family of lower bounds several of which are close to the true reliability under reasonable conditions. Finally, we argue that eventually sum scores derive their value from the degree to which they enable predicting practically relevant events and behaviors. None of our discussion is meant to discredit modern measurement models; they have their own merits unattainable for classical test theory, but the latter model provides impressive contributions to psychometrics based on very few assumptions that seem to have become obscured in the past few decades. Their generality and practical usefulness add to the accomplishments of more recent approaches.

Many psychometricians have banned the sum score to psychometrics’ mausoleum, where it rests among concepts once thought useful but later replaced with alternatives holding greater promise. Braun and Mislevy (2005) coined the term *Intuitive Test Theory* for ideas that are useful at the level of having a sense of how things work but without the up-to-date knowledge of how phenomena really function. This up-to-date knowledge is part of *Scientific Test Theory*. On their list of ten *phenomenological primitives* or *p-prims* (diSessa, 1993) is “You Score a Test by Adding up Scores for Items”. They explain that the sum score misses important information when tasks are complex requiring several skills and abilities or when one is interested in how students solve problems rather than whether they solve them correctly. According to Braun and Mislevy, Scientific Test Theory involves probabilistic models that relate observed responses to items provided by respondents to unobservable (i.e., latent; *the authors SEB*) variables that are more convenient for the assessment of performance on tests and questionnaires. Although they do not claim that sum scoring is always inferior to scoring or assessment methods that are more nuanced, putting it on the list of p-prims includes it in Intuitive Test Theory. Mislevy (personal communication) notes that the sum score belongs to Intuitive Test Theory when it is used without scientific justification but that it belongs to Scientific Test Theory when scientific arguments justify this. In this contribution, we provide such arguments at a general level.

After more than a century, many psychometricians are struggling with the sum score as a quantification of an individual’s performance on a test or a questionnaire and many reject it in favor latent-variable scores scores implied by item response theory (IRT) models. For example, Borsboom (2005) has argued that, from a realist perspective on measurement, the sum score can be understood as a measure of an attribute, such as spatial-orientation ability or extraversion, only if that attribute acts as a common cause of the item responses that make up the sum score, which means that, from this perspective, its measurement properties depend implicitly on a latent-variable model that adequately explains the item responses. Holland and Hoskens 2003 similarly view inferences based on the sum score in classical test theory (CTT) as a first-order approximation of the item response theory (IRT) model. Although these lines of thought do not disqualify the use of sum scores, they do contribute to the view that the use of sum scores constitute a poor man’s version of psychometrics.

In a more extreme articulation of this idea, McNeish and Wolf (2020a; b) provided a highly critical account of the sum score; from their point of view, the sum score should simply be abandoned. Widaman and Revelle (2022) have countered their line of reasoning in defense of the sum score; also, see McNeish (2023) and Widaman and Revelle (2023) for a continued discussion. The point of view expressed by McNeish and Wolf (2020a, b), McNeish (2023) and in similar phrasings by many other authors introduces a second criticism of the sum score, predominantly voiced in the context of the factor analysis (FA) model. The idea then is that only if the sum score is represented in a restricted 1-factor model explanation of the item responses will CTT facilitate the unbiased estimation of sum score reliability, thus introducing reliability into the discussion about the sum score. Because the critics consider a restricted 1-factor model unrealistic as an explanation of item responses, they conclude that CTT will fail, and based on that conclusion they claim reliability estimation must adopt the FA model in versions that explain the item responses in particular instances.

These two criticisms, which are that the IRT latent variable is superior to the CTT sum score and that CTT is too restrictive to provide reliability estimation methods unbiased for real item responses, have made life difficult for the simple sum score, as is witnessed in many recent publications. In this contribution, we argue that, even though there are certainly cases in which the uncritical use of sum scores is suboptimal, this does not mean that the sum score is generally useless. In contrast, we show that the sum score possesses some highly desirable properties and that, in some instances, it can be superior to more advanced ways of scoring a test (e.g., through latent-variable models). In addition, we show that, while it is true that the fit of a unidimensional model (e.g., the 1-factor model) can support the interpretation of test scores, the fit of such a model is not necessary for the productive use of sum scores. In arguing this case, we will show that both objections suffer from critics’ tendency to be dismissive of an older test theoretical model, in this case, CTT, while they do not subject their preferred models, in our discussion either IRT or FA, to the same level of scrutiny. In doing so, critics tend to overlook certain results contradicting or at least mitigating their criticisms, and in the worst case, they may even try to find arguments in favor of a foregone conclusion, abandonment of the sum score and CTT-based reliability.

In discussing the topic before us, we isolated the two criticisms as central in a discussion on the sum score and concluded the sum score and CTT need a reappraisal. Hence, in this contribution we discuss the usefulness of the sum score for ordering people with respect to an attribute scale and the correct use of CTT for estimating sum-score reliability. We notice two issues in advance. First, because the psychometric literature has grown so huge and with it the number of critical papers, it is hopeless to try discussing all contributions that are related to the two topics we discuss here. Thus, we limit ourselves to what we consider the most relevant references. Second, psychometrics is plagued by misunderstandings that cloud, confuse, or even misdirect the discussion about the sum score. We will touch upon such misunderstandings occasionally and try to identify them, but we have refrained from an exhaustive discussion.

The structure of this paper is as follows. First, we show that in the IRT context the sum score in principle can both be used as an approximation or estimate of the latent variable. In addition, we discuss some first results of using sum scores in network models. Second, we discuss a general formulation of CTT and argue that it is a general theory of measurement error or noise. Unlike IRT and FA models and contrary to popular belief, CTT does not restrict the dimensionality of the test and can be used in situations where the items do not measure the same attribute. Acknowledging its limitations, we will also point out the elegance of CTT and the lower-bound methods for reliability estimation it provides in such cases. Finally, we provide a few take-home messages in the Discussion section. Throughout, we focus on standard tests where each person receives the same set of items and we refrain from larger-scale testing programs involving equating of various scales and adaptive testing.

## The Sum Score and the Latent Variable: Two Sides of the Same Coin?

In this section, we discuss the relation between the sum score and the latent variable as we know it from IRT. CTT and IRT are the main current psychometric theories that are used to construct and analyze psychological and educational tests. Both utilize the sum score, but they do so in different ways. In the current section, we examine the relation between these theories and the role the sum score plays in each of them. We will assume that the reader is familiar with both CTT and IRT, but because we noticed that knowledge of CTT in general is subject to wear and tear, later we will discuss CTT in more detail, thus justifying the reliability results that often give cause to discussion.

### The Sum Score and the Latent Variable in IRT

A test consists of *J* items. Items are indexed *j*, so that $j=1,\cdots , J$. The score on an item is denoted $X_{j}$. For reasons of simplicity, we assume that integer item scores run from 0 to *m*; if $m=1$, then the item is dichotomously scored, and if $m>1$, then it is polytomously scored. The sum score is defined as

$$\begin{aligned} X_{+}=\sum \limits _{j=1}^J X_{j}. \end{aligned}$$

Here, we need a few definitions in the context of CTT, anticipating a deeper discussion of CTT in the next section. At the level of items, we define the CTT decomposition,

$$\begin{aligned} X_{j}=T_{j}+E_{j}, \end{aligned}$$

where score component $E_{j}$ correlates 0 with the score component $T_{j}$, as well as with the score components $E_{k}$ and $T_{k}$ of all other items in the test. So, in addition to the definition of the sum score we define the true score of the sum score as

$$\begin{aligned} T_{+}=\sum \limits _{j=1}^J T_{j}. \end{aligned}$$

Score components $E_{j}$ are typically interpreted as random measurement errors. It is important noticing that although item true scores are specific to the item, this does not preclude that some or even all items measure the same attribute. At the other extreme, each item may measure a completely different attribute. While this circumstance may be considered unlikely in practical test construction, where researchers aim at a set of items that measure a common attribute, it is important to note that CTT does not assume or require this. CTT allows for every possibility.

Test scores, as used in practice when all persons receive the same items, are typically transformations of the sum score. Such transformations may take many forms, such as (normalized) standard scores, stanines, percentiles, and IQ-scores. Because the sum score, often called the raw score as well, is usually at the basis of test scores, we focus on the sum score without loss of generality of the conclusions we draw, ignoring possible exceptions which will not undermine the points we wish to make. The sum score has a long history going back at least to Binet and Simon (1905). For a long time, even until today, its reliability and validity have been subject of investigation, where CTT is vital for reliability estimation and together with a wide array of multivariate methods, mostly regression modeling and factor analysis, also for validity research.

Dating back to the 1930 s (Richardson, 1936), 1940 s (e.g., Brogden, 1946; Ferguson, 1942; Finney, 1944; Lawley, 1943) and 1950 s (e.g., Lord, 1952; Cronbach & Warrington, 1952), IRT started flourishing in the 1960 s and 1970 s and gained an increasing popularity since the 1980 s in psychometrics where it pushed aside CTT. In the practice of test and questionnaire construction outside large educational testing organizations like Educational Testing Service, this effect was less pronounced; here, CTT and the accessible sum score held their dominant position for a long time until today. Psychometricians appreciated IRT’s improvements to CTT, a circumstance Lord (1980) confirmed in his seminal book but with the caveat (ibid., p. 7) that “nothing in this book will contradict either the assumptions or basic conclusions of classical test theory,” a truth ignored regularly in much of psychometrics. What precisely are these improvements? We think an important improvement relative to CTT was that IRT added restrictions on the dimensionality of the test score. In this sense, IRT is comparable to modern versions of FA (e.g., Bollen, 1989). In both models, the score for item *j* is a function of one or more latent variables that the whole set of *J* items share.

IRT models are nonlinear. For example, for the unidimensional 2-parameter logistic model for dichotomous items (e.g., correct/incorrect or 1/0 scoring), the score on item *j* is modeled by the increasing item response function,

$$\begin{aligned} P\left( {X_{j}=1} \vert \theta \right) =\frac{\exp [\alpha _{j}\mathrm {(\theta -}\delta _{j}\mathrm {)]}}{1+\exp [\alpha _{j}\mathrm {(\theta -}\delta _{j}\mathrm {)]}}, \end{aligned}$$

with $\theta$ being the latent variable common to the *J* items, $\alpha _{j}$ the slope or discrimination parameter, and $\delta _{j}$ the location or difficulty parameter. Many different IRT models have been proposed for dichotomous item scores and polytomous item scores, unidimensional and multidimensional latent structures, different mathematical response functions, parameter decompositions, and various other possibilities; see Van der Linden and Hambleton (1997), Van der Linden (2016), and Sijtsma and Van der Ark (2021) for extensive overviews.

Considering IRT models, it is understandable why they appealed more to psychometricians than CTT—because of their greater complexity, they provide more possibilities for instrument construction than CTT alone does. It is interesting that monotone unidimensional IRT models like the 2-parameter logistic model, restrict the correlations between the *J* items to be positive (Holland and Rosenbaum, 1986; Ligtvoet, 2022) whereas CTT does not imply this restriction due to the absence of dimensionality restrictions; recall that each item is allowed to have its unique true score. CTT has no dimensionality restrictions, but on the other hand, it does not exclude dimensionality restrictions either. Thus, CTT contains models with dimensionality restrictions as special cases, a situation that readily invites confusion as we will see when we discuss reliability in the next section.

An early IRT model that gained much popularity in the 1960 s to 1980 s in Australia and Europe and less in the USA was the 1-parameter logistic model or Rasch model (Rasch, 1960, 1968). The defining characteristic of the model is that the sum score $X_{+}$ is a minimally sufficient statistic for the estimation of the one common latent variable the Rasch model assumes. In nonstatistical terms, this means that the sum score contains all the information needed for estimating the latent variable and that one can ignore which items a person succeeded or failed. This important property should give any critic of the sum score pause for thought.

In fact, Fischer (1974, 1995) *derived* the Rasch model from the assumption that the sum score $X_{+}$ should be sufficient for the estimation of $\theta$, together with the assumptions of unidimensionality (one latent variable $\theta )$, conditional independence (the *J* marginal item-score distributions given $\theta$ imply the joint distribution given $\theta )$, and monotonicity (the higher $\theta$, the higher the probability of obtaining a score of 1—correct or affirmative response—on the item), and differentiable item response functions with range (0, 1). Under these assumptions, the item response function which defines the Rasch model for item *j* can be derived to equal,

$$\begin{aligned} P\left( {X_{j}=1} \vert \theta \right) =\frac{\textrm{exp}(\theta -\delta _{j})}{1+ \textrm{exp}(\theta -\delta _{j})}, \end{aligned}$$

and is monotonically increasing in latent variable $\theta$. Inserting $\alpha _{j}=1$ in the equation for the 2-parameter logistic model yields the equation for the Rasch model.

Since $\mathbb {E} (X_{j}\vert \theta )=P\left( {X_{j}=1} \vert \theta \right)$, we alternatively define the true score on item *j* as

$$\begin{aligned} T_{j}(i)=\mathbb {E} (X_{j}\vert \theta =\theta _{i})=P\left( {X_{j}=1} \vert {\theta =\theta _{i}}\right) . \end{aligned}$$

For a randomly selected $\theta$ value, the true score on item *j* is denoted $T_{j}$. The test response function is defined as the sum of the item true scores, so that,

$$\begin{aligned} T_{+}=\sum \nolimits _{j=1}^J T_{j}. \end{aligned}$$

**Fig. 1:**
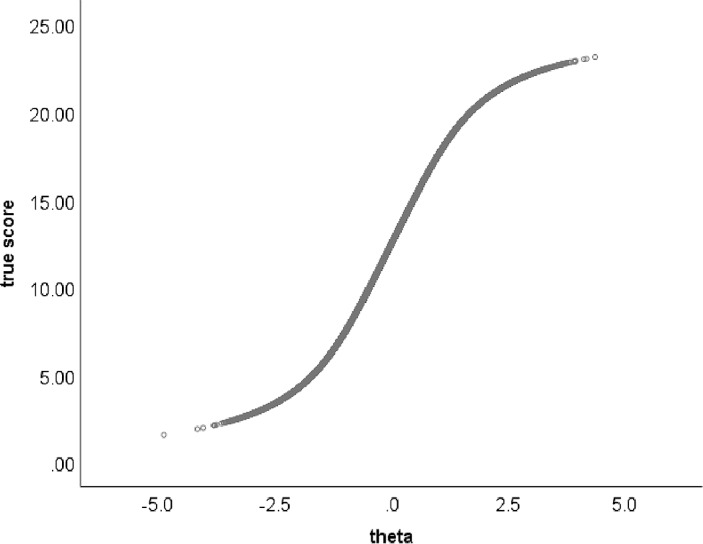
Test response function for a *J*-item test based on the 2-parameter logistic model.

Figure 1 shows a test response function for a *J*-item test based on the 2-parameter logistic model. Because each item response function is monotonically increasing, the relationship between latent variable $\theta$ and true sum score $T_{+}$ is also monotonically increasing. Moreover, depending on the choice of the item parameters, the test response function approximates linearity in the middle of the $\theta$ distribution, while nonlinearities appear in the tails of the distribution. One can manipulate the location of the items to produce test response functions that deviate more from linearity, but these choices are not typically encountered with real items. Here, what matters especially is that the latent variable and the true sum score are monotonically related under the assumption that the latent-variable model holds, a result that generalizes to other monotone IRT models as well, for both dichotomous and polytomous item scores (Sijtsma & Van der Ark, 2021).

Given the monotone relationship between the latent variable and the true sum score, it is surprising how little attention psychometricians studying the Rasch model granted the true sum score and its estimate, the sum score, a circumstance applying to IRT theorists in general. The minimal sufficiency property of the sum score in the Rasch model rather played a role as a steppingstone to the more appreciated latent variable and its statistically well-founded estimate, which left no room for the true sum score and its estimate, the sum score. However, Fig. 1 already hints at the close relationship between the latent variable and CTT’s true sum score, suggesting the difference may not be that great and even raising the issue whether one should prefer a less well interpretable latent-variable estimate to a true sum-score estimate—the sum score—that researchers and laypeople experience as less alienating and better suited for communication of test results (Hemker, 2023). Clearly, a psychometrician who appreciates IRT need not be dismissive of the sum score. Instead, one can view the IRT model as furnishing an important *justification* for the use of the sum score, because if the IRT model holds, we know that the sum score has the important property of being monotonically related to the latent variable. This property remains true for item subsets and equivalently, different item sets constituting different scales but each measuring the same attribute.

For a long time, it appeared as if the sum score could only be used if a Rasch model holds, because the sufficiency property does not typically apply to other IRT models. As a result, because the restrictive Rasch model rarely fits item response data adequately, many psychometricians abandoned the Rasch model (or never supported it) in favor of more flexible IRT models such as the 2- and 3-parameter logistic models (Birnbaum, 1968) and their normal-ogive versions (Lord, 1952, 1980). The same is true of the polytomous-item Rasch model and more flexible models for polytomous items. Since then, the number of IRT models grew at an astonishing speed (e.g., Van der Linden & Hambleton, 1997; Van der Linden, 2016; Sijtsma & Van der Ark, 2021). The sum score lost ground quickly, and its decline was not interrupted by a paper (Grayson, 1988) that might have opened some eyes were it not that it was largely ignored.

### General IRT and the Sum Score

The value of Grayson’s (Grayson, 1988; also, see Hemker et al., 1996, 1997; Huynh, 1994; Junker, 1993; Unlü, 2008; Van der Ark, 2005; Van der Ark & Bergsma, 2010) contribution is considerable: Grayson proved that for binary items, under milder assumptions than those of the Rasch model, the 2- and 3-parameter logistic models, and less restrictive models, the sum score and the latent variable have monotone likelihood ratio. This means that for all $x_{+1}$, $x_{+2}$ with $0\le x_{+1}<x_{+2}\le J$, the ratio $P\left( {X_{+}=x_{+2}} \vert \theta \right) /P(X_{+}=x_{+1}\vert \theta )$ is a nondecreasing function of $\theta$. Hemker et al. (1996) pointed out that this implies that the posterior distributions of the latent variable given the sum score are consistently ordered in the sense that $P(\theta \le t\vert X_{+})$ is decreasing in $X_{+}$ for each *t* in the range of $\theta$. Hemker et al. (1997) call this property stochastic ordering of the latent variable (SOL). We focus on this property in this section because it is easier to interpret than the stronger monotone likelihood property. Importantly, this established that highly favorable measurement properties of the sum score are not limited to the Rasch model but generalize to many other models in the IRT family.

Stochastic ordering refers to a property of cumulative distributions, but we start with the implication for latent variable means that, when conditioned on the sum score, these latent variable means increase as the sum score increases. Therefore, the discrete sum score can be viewed as a robust ordinal approximation of the continuous latent variable. Thus, Grayson provided a new *raison d’être* for the sum score, now based on IRT assumptions, and not simply assumed as in CTT. To enhance its recognition, we will discuss the stochastic ordering property differently from the mathematical treatments given in the relevant literature. Figure 1 may help to attain our goal but first we notice that it is based on the assumptions of unidimensionality (one latent variable $\theta )$, conditional independence (of the joint distribution of the items given $\theta )$, and monotonicity (item response functions nondecreasing in $\theta )$, but not on the sufficiency of the sum score $X_{+}$ for the estimation of $\theta$, which is the fourth assumption of the Rasch model. Item response functions thus can have any functional form provided that the function is monotone, meaning that it does not show any local decreases. This model is known in the psychometric literature under various names (e.g., Ellis & Sijtsma, 2023). We use the name of monotone homogeneity model (Sijtsma and Molenaar, 2002), because under this name the model has been used the most for scale construction.

**Fig. 2:**
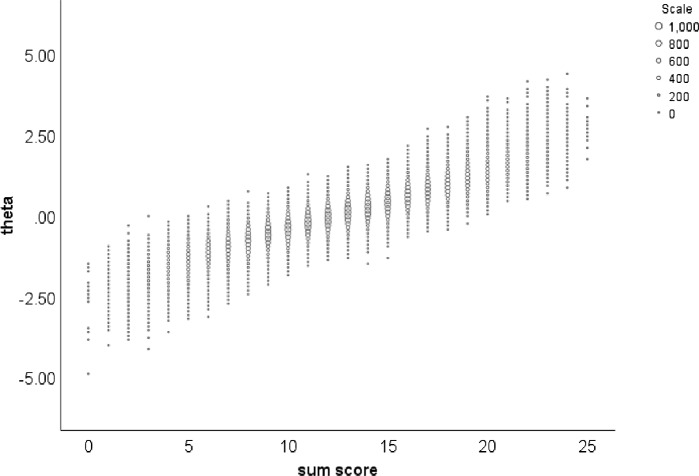
Distribution of latent variable $\theta$ conditional on sum score $X_{+}$ assuming the 2-parameter logistic model.

The 2- and 3-parameter logistic models, their normal-ogive versions, and other monotone unidimensional IRT models having more item parameters are all special cases of the monotone homogeneity model. Because the monotone homogeneity model formulation involving $P(X_{j}=1\vert \theta )$ only restricts this response function ordinally but does not define a parametric monotone function including parameters that might be estimated from the model’s likelihood or via a Bayesian procedure, it does not enable numerical estimation of $\theta$ from the data. Instead, for the special case of the 2-parameter logistic model, chosen only because it facilitates computation, Fig. 2 shows a pattern that is generally correct for the monotone homogeneity model and all its special cases. (The figure is based on $J=25$, location parameters between $-2$ and 2, slope parameters between 0.1 and 2.5, and $\theta \sim \mathcal {N}(0, 1)$ with ${100,{\!}000}$ random draws, and shows the conditional distributions of $\theta$ given sum score $X_{+}$, $f(\theta \vert X_{+})$.)

The fascinating observation concerning the pattern is that as the sum score increases, the conditional distribution of the latent variable, $f({\theta }\vert X_{+})$, moves up and it does this in a most interesting way, but first we notice that the means of the distributions also increase. For the population case, we consider two realizations of $X_{+}$ and call them $x_{+v}$ and $x_{+w}$, and assume arbitrarily that $0\le x_{+v}<x_{+w}\le J$. Then,

$$\begin{aligned} \mathbb {E} (\theta \vert {X_{+}=x}_{+v})\le \mathbb {E} (\theta \vert {X_{+}=x}_{+w}), \text { all } x_{+v}<x_{+w}, \end{aligned}$$

and Fig. 2 shows the result for a large sample approximating the population. The meaning of this result may not be immediately clear. It is that if we divide the population into subpopulations each having the same sum score $X_{+}$ and then order them by increasing sum score, we have also ordered them by increasing mean latent variable in the homogeneous sum-score subpopulations. The meaning is that the regression of $\theta$ on $X_{+}$ is monotone increasing, which complements the earlier conclusion that the regression of $X_{+}$ on $\theta$ is monotonically increasing (the test response function of Fig. 1).

Thus, the monotone homogeneity model implies that the sum-score ordering and the ordering by mean latent-variable score are equal except for random error in the sum score, and that sum scores yield consistently ordered posterior distributions of the latent variable. It is worthwhile realizing this is true even if one cannot estimate the latent variable: The latent variable itself remains unknown but ordering groups that are homogeneous with respect to the sum score by the sum score guarantees ordering by mean latent variable. Like the latent variable, the true score is continuous but its estimate, the sum score is discrete. Figure 1 shows the test response function and by interchanging the two axes we have the inverse relationship mapping the true score (abscissa) on the latent variable (ordinate). The point is that the relationship is a monotone curve without scatter representing a conditional distribution. Figure 2 shows that replacing the true score on the abscissa with the coarser sum score implies that the monotone curve relating true score and latent variable is replaced with $J+1$ conditional distributions of which the means are increasing with the sum score.

**Fig. 3:**
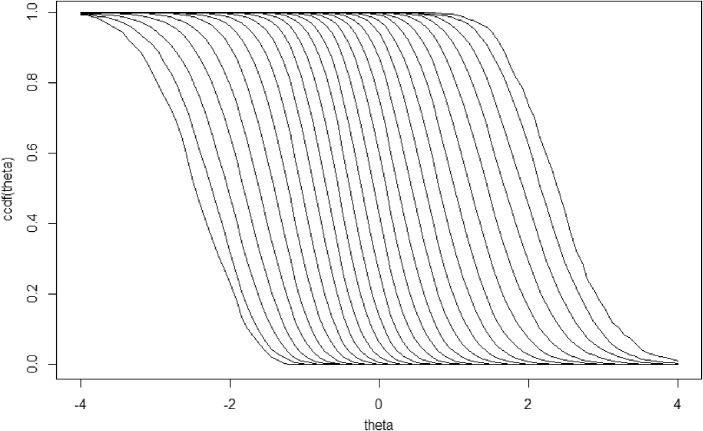
The 26 complementary cumulative distributions, $F(\theta \vert X_{+})$, corresponding to the distributions $f(\theta \vert X_{+})$ in Fig. 2. Based on $N={10}^{6}$ to have sufficient precision.

Returning to the conditional distributions in Fig. 2 and wondering what is special about their ordering, we must consider the cumulative distributions, $F(\theta \vert X_{+})$, or better, the complementary cumulative distribution, $1-F\left( \theta \vert X_{+}\right) =P(\theta >t\vert X_{+})$, where *t* is a realization of $\theta$. Figure 3 shows these complementary cumulative distributions corresponding to the conditional distributions in Fig. 2. What they show is the property of SOL (Hemker et al., 1997),

$$\begin{aligned} P(\theta>t\vert {X_{+}=x}_{+v})\le P(\theta >t\vert X_{+}=x_{+w}), \text { all } t,\text { all } x_{+v}<x_{+w}. \end{aligned}$$

What does SOL mean? Figure 3 shows the 26 complementary cumulative distributions, $F({\theta }\vert X_{+})$, corresponding to the distributions $f({\theta }\vert X_{+})$ in Fig. 2. We see that SOL means that the complementary cumulative distributions shift to the right as sum score $X_{+}$ increases, even though their shape may change somewhat but never enough to force intersecting curves. Hence, SOL means that as the sum score $X_{+}$ increases, more fixed latent variable scores $\theta$ from $f(\theta \vert X_{+})$ exceed a fixed value $\theta =t$; not just one *t*-value but any *t*-value! For a fixed sum score $X_{+}$, the proportion $P(\theta >t\vert X_{+})$ decreases as *t* increases. Another way of expressing the latter result is that a higher bar *t* makes it more difficult to jump over it so that a smaller proportion of the subgroup with fixed $X_{+}$ succeeds.

This result is just what one would expect of the sum score, and it should be reassuring to psychometricians and researchers that by using IRT models they can justify the use of the intuitively so much more appealing and transparent sum score.

The theory-based, confirmatory approach justifying a monotone unidimensional measurement model implies that an acceptable fit of the monotone homogeneity model or more specific IRT models to the data facilitates ordering people according to their sum scores, which in turn implies ordering them according to the mean latent variable in the sum-score group, but without the necessity of estimating the latent variable.

The reader might tend to believe that an estimator of $\theta$ that is optimized according to some explicit statistical criterion, such as a maximum likelihood estimator, is always superior to the “one size fits all” simple sum score, since the latter does not fully capitalize on the information in the data. However, in the derivation of the optimization it is usually assumed that the item response functions are completely known. That is, the shapes of the item response functions should be completely specified (e.g., logistic), and the specification should be entirely correct while the item parameters should be known exactly. We do not know of a realistic situation in which these requirements are satisfied. Even if the model is correctly specified, the item parameters are always estimated from a finite sample, and therefore not known exactly. Therefore, we expect that the presumed superiority of formal estimators of $\theta$ may fail if the item response functions are incorrectly specified or if the sample size is small.

We studied the latter situation using a simulation study in which we generated data using the 2-parameter logistic model, computed the sum score $$X_{+}$$ for each person, and estimated the latent variable $$\theta $$ using weighted maximum likelihood (WML; Warm, 1989; software package mirt, Chalmers, 2012) resulting in $$\hat{\theta }$$, repeated the procedure 100 times, and for each data set computed the correlations $$r_{1}=r(X_{+},\theta )$$ and $$r_{2}=r(\hat{\theta },\theta )$$. We compared $$r_{1}$$ with $$r_{2}$$ to determine whether $$X_{+}$$ or $$\hat{\theta }$$ was the better predictor of $$\theta $$. The choice of true $$\theta $$ as the criterion to be predicted maximally favors estimate $$\hat{\theta }$$ and puts $$X_{+}$$ at a disadvantage; thus, we used the criterion that favors the opponents of our claim that the sum score wins. We studied this for various ranges of item parameters, sample sizes, test lengths, and model misspecifications. In many cases, we found that $$X_{+}$$ was superior to $$\hat{\theta }$$ in the sense that $$r_{1}>r_{2}$$ in most of the samples. Specifically, in some cases $$r_{1}$$ was somewhat larger than $$r_{2}$$ but in other cases, $$\hat{\theta }$$ ($$r_{2})$$ never blew away $$X_{+}$$ ($$r_{1})$$ as a predictor of $$\theta $$, as one might have expected. For violations of logistic item response functions, for various sample sizes and test lengths, we often found $$r_{1}>r_{2}$$ with quite large mean differences. Appendix A gives detailed results. Two additional comments with respect to SOL are the following. First, for polytomous-item IRT models, ordering properties such as SOL do not hold analytically (Hemker et al., 1997), but Van der Ark (2005) provided mitigating circumstances by demonstrating, by means of data simulated with a variety of IRT models for polytomous items, that ordering persons by means of their sum scores usually results in an ordering by latent-variable scores that either reflects the correct ordering (in most cases) or only shows minor deviations concerning (nearly) neighboring scores, so that no grave ordering errors are made. In addition, Van der Ark and Bergsma (2010) proved analytically that, for a wide range of polytomous-item IRT models, a weaker ordering property than SOL holds for a division of the total group into two subgroups, one containing only lower sum scores and the other only higher sum score (e.g., defined by $$X_{+}\le x_{+}$$ and $$X_{+}>x_{+})$$. Ligtvoet (2022b) showed that the sum score stochastically orders the factor in the linear normal 1-factor model. Second, Stout (1990, p. 309, theorem 3.2) proved ordering properties for an IRT model based on assumptions that are even weaker than those on which the monotone homogeneity model is based and of which almost all unidimensional parametric IRT models are special cases. He proved that his model of essential unidimensionality for dichotomously scored items enables that “the number correct score consistently estimates ability on the latent true score scale” (Stout, 2002, p. 491). In this quote, total test score and number correct score are equivalent with the sum score. The latent true-score scale refers to the mean of the sum of the response probabilities, $$\sum \nolimits _{j=1}^J {P\left( {X_{j}=1} \vert \theta \right) /J} $$, which we recognize as the test response function (Fig. 1) and which is nondecreasing in $$\theta $$, with the difference with previous models that individual item response functions may be nonmonotone. Now assume that the test response function is strictly increasing and that its derivative exists and is bounded away from 0 ( (Stout used a slightly weaker assumption than this, but the difference is not important here). The consistency Stout described means that if we set $$\bar{X}_{J}=\sum \nolimits _{j=1}^J {X_{j}/J} $$ and $$\bar{T}_{J}(\theta )=\sum \nolimits _{j=1}^J {P\left( {X_{j}=1} \vert \theta \right) /J} $$ and $$\bar{T}_{J}^{-1}$$ is the inverse of $$\bar{T}_{J}$$, then $$\bar{T}_{J}^{-1}(\bar{X}_{J})$$ converges in probability to $$\theta $$. That is, if the sum score is properly transformed, then the transformed sum score goes to $$\theta $$. It is customary to assume a continuous distribution for $$\theta $$, and since ordinal scale transformations are admissible in this model, we may choose a scale version of $$\theta $$ that has a uniform distribution on (0, 100). With this scale of $$\theta $$, our interpretation of Stout’s theorem is that under said conditions of essential unidimensionality and derivatives bounded away from zero, the percentile ranks of the sum score converge to $$\theta .$$ We call this *ordinal consistency* of the sum score. We refrain from discussing other relaxations of assumptions of the monotone homogeneity model. Stouts’ result is remarkable because it is obtained under a general class of IRT models which do not even need to be unidimensional and conditionally independent, and have a mean item response function—a test response function—that is monotone without imposing monotonicity on individual item response functions, while including 1-, 2-, and 3-parameter logistic and normal-ogive models as well as nonparametric models assuming monotone functions that may or may not intersect. Junker (1991) generalized the result of Stout to items with polytomous scores. We conclude that “You Score a Test by Adding up Scores for Items” may have been intuitive at the start of psychometrics more than a century ago but also note that, if based on intuition, it proved a highly fortunate hunch that has been substantiated through a century of psychometric theory formation. The sum score is not just a poor man’s test score but provides a defensible ordinal approximation of the latent variable under surprisingly general conditions. This claim is supported by investigations concerning a wide range of IRT models, in each of which the sum score stochastically orders the latent variable (or closely approximates the stochastic ordering) defined in these models. This gives the sum score a solid mathematical basis and justifies its use in measurement practices. In an even wider range of essentially unidimensional IRT models, the sum score is a consistent ordinal estimator of the latent variable, as explained previously.

### Network Models and the Sum Score

Both CTT and IRT have traditionally been developed and applied in situations in which researchers think of psychometric attributes such as intelligence, depression, or attitudes as latent attributes that determine people’s responses to specific items, such that the latent variable in an IRT model plays the role of a common cause of the item responses (e.g., see Bollen, 1989; Bollen & Pearl, 2013; Van Bork, Rhemtulla, Sijtsma, & Borsboom, 2022). While such a causal interpretation should not be seen as part of the statistical axioms that characterize the model, it does provide a sensible reason for using it: If one believes that the item responses depend on the same psychological attribute, then it makes sense to test that hypothesis with a latent-variable model, and if that model is adequate this provides a justification for the interpretation of the test score as an estimate of the latent variable.

However, analyses of psychological attributes like general intelligence (van der Maas et al., 2006), depression (Cramer et al., 2016), and attitudes (Dalege et al., 2016) have suggested an alternative point of view, in which the correlation between observables arises from direct causal interactions in a complex system. For instance, in the case of depression, symptoms like insomnia, fatigue, and concentration problems may at least in part result from direct interactions (e.g., insomnia can lead to fatigue and concentration problems) rather than from a dependence on a common latent variable (Borsboom & Cramer 2013). In such cases, network models have been proposed as an alternative psychometric representation of the relation between constructs and observables (Marsman and Rhemtulla, 2022).

An interesting question is what the status of the sum score is in such models. One possible way of addressing this issue is by examining the use of sum scores in models taken from statistical mechanics, such as the Ising model (Marsman et al., 2017). Such models concern the question how a conglomerate of interacting components (in this case, particles) behaves at the macro-level; for instance, how does magnetism arise from interactions between ferromagnetic particles and how does pressure arise from collisions between the atoms that make up a gas. In such cases, it turns out that the average state of the components of the system is routinely used to approximate the global behavior of the model as a first-order (i.e., the mean-field approximation; Finnemann, Borsboom, Epskamp, & Van der Maas, 2021). Of course, for a system of a given number of components, the average state of the components is a linear transformation of the sum score. This suggests that the sum score may be useful for tracking the global behavior of a network (Van Bork, Lunansky, & Borsboom, 2024).

In such cases, the sum score need not be interpreted as a measurement of a latent variable. Alternatively, it can be thought of as an index, which can be used to assess the overall state of the system (Van der Maas, Kan, & Borsboom, 2014). Such indices are used throughout the sciences. Outside of psychometrics, for instance, similar applications are the so-called AEX index, which expresses the value of the total Amsterdam Stock Exchange based on the stocks of the 25 biggest companies freely available for trading, and the *h*-index (Hirsch, 2005) expressing a researcher’s productivity in combination with impact. Although not based on IRT or other models, a large array of validated psychological tests derive their usefulness from their ability to track or predict other indices or behaviors considered useful from a practical rather than a psychometric point of view, by using the sum score.

To illustrate this point, we can investigate how the use of the sum score would fare in the well-known Ising model (Ising, 1925). In the Ising model, the behavior of a system is modeled as a function of symmetric pairwise interactions between its components, which can be in one of two states (e.g., 0 or 1). These interactions are commonly represented in a network, in which each component is a node, and each interaction is an edge. Each of the *J* components follows a probability distribution which is controlled by two factors: the autonomous tendency of the state variable $X_{j}$ for component *j* to take the value 1 (known as a threshold $\tau _{j})$ and the state of other components to which *j* is connected through a set of interaction parameters (known as edge weights $\omega _{jk}$that represent the strength of the interaction between nodes *j* and *k*).

The configuration of the states of all components defines a random vector $\mathrm {\textbf{X}}$, taking on values$\mathrm {\textbf{x}}$. The Ising model then represents the probability of any configuration $\mathrm {\textbf{X}}=\mathrm {\textbf{x}}$, given a vector of thresholds ${\varvec{\uptau }}$ and a matrix of edge weights ${\varvec{\upomega }}$, as:

$$\begin{aligned} P\left( {\mathrm {\textbf{X}}=\mathrm {\textbf{x}}} \vert \varvec{\uptau },\varvec{\upomega }\right) =\frac{\textrm{exp}(-\beta H\left( \mathrm {\textbf{x}} \right) )}{Z}. \end{aligned}$$

In this equation, $\beta$ is a so-called temperature parameter that controls how much randomness the system exhibits. In the present context, this parameter does not matter, and we can set it to unity without loss of generality. *Z* acts as a normalizing constant, as it equals the sum of the values $-\beta H\left( \mathrm {\textbf{x}} \right)$ over all configurations (Finnemann et al., 2021). The second summation, $<j,k>$, is across all combinations of *j* and *k*, with $j\ne k$. The function $H\left( \mathrm {\textbf{x}} \right)$ is known as the Hamiltonian and has the form,

$$\begin{aligned} H\left( \mathrm {\textbf{x}} \right) =-\sum \limits _j {\tau _{j}x_{j}} -\sum \limits _{<j,k>} {\omega _{jk}x_{j}x_{k}.} \end{aligned}$$

Because the Ising model generates a distribution over all states $\mathrm {\textbf{x}}$, it also generates a distribution of the sum score variable $X_{+}$. The summation, $\mathrm {\textbf{x}}\mathbf {\rightarrow }x_{+}$, is across all vectors $\mathrm {\textbf{x}}$ of which the elements sum to $x_{+}$. This distribution has the form:

$$\begin{aligned} P(X_{+}=x_{+}\vert \varvec{\uptau },\varvec{\upomega })=\sum \limits _{\mathrm {\textbf{x}}\mathbf {\rightarrow }x_{+}} {P\left( {\mathrm {\textbf{X}}=\mathrm {\textbf{x}}} \vert {\mathrm {\varvec{\uptau }},\mathrm {\varvec{\upomega }}}\right) .} \end{aligned}$$

Thus, the probability of observing a given sum score equals the sum of the probabilities of the network states that produce this sum score.

This type of system may be taken to characterize the network of an individual person *i*. Similar to CTT, that person would thus be characterized by a probability distribution of the sum score as given above (cf. a propensity distribution; Lord & Novick, 1968, 1974). Any observation of the system, which can be conceptualized as a sample from the distribution, constitutes an observed sum score. Analogous to the CTT setup, for the individual we may then break up this sum score into a true network score for person *i*, which we may take to equal $\mathbb {E} (X_{+}\vert person=i)$, and a random component, which we may define as $X_{+}^{(i)}-\mathbb {E} (X_{+}\vert person=i)$. This is exactly parallel to the setup in Lord and Novick (1968, 1974) but note that the individual components need not correspond to any IRT model. Although there generally will be some statistically equivalent multidimensional IRT model (Epskamp et al., 2016; Marsman et al., 2018) that describes the system, the interaction matrix of edge weights may feature zeros (conditional independencies), negative entries (violations of positivity), and clustering (violations of unidimensionality).

**Fig. 4:**
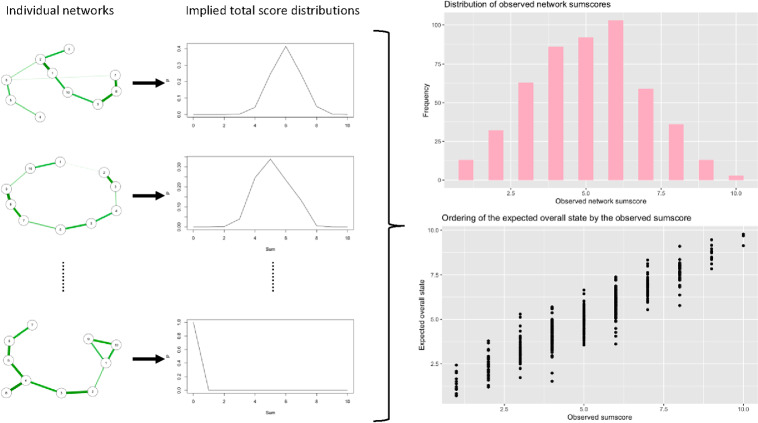
Results of the network simulation. Different network structures are generated for each individual, after which the implied distribution of the sum score for these networks is determined (left and middle panel). The expected value of this distribution characterizes the expected overall state of the network. The statistical relation between the expected overall states and observed sum scores suggests a stochastic ordering relation (right panel).

Now suppose that individuals differ in the structure and parameterization of their networks. We may, for instance, imagine a small attitude network (Dalege et al., 2016) representing the attitude toward, say, organ donation. Suppose the network contains three nodes that represent a cognitive state (e.g., “organ donation is useful”), an affective state (e.g., “organ donation is scary”), and a motivational state (e.g., “I want to be a donor”). Individuals may differ in the tendency of these nodes to take a value (e.g., John may be an apprehensive person who finds things scary, while Mary is not easily scared, representing a different threshold $\tau$ for the affective node) and the tendency of the nodes to align (e.g., John’s affective node may have a stronger influence on his behavior than Mary’s, representing a stronger interaction parameter $\omega$ between the affective and behavioral nodes). Given such individual differences in network structure, there will be individual differences in the true network score. We can take this true network score to represent the expected overall state of the system, and this may be a plausible target for measurement in a network context (Van Bork et al., 2024). Of course, on any specific assessment occasion, we only obtain an observed sum score. Hence, as in CTT and IRT, we can now ask the question: How well will the observed total score track the true network score in this context?

Although a detailed mathematical investigation of this question is beyond the scope of this discussion, a tentative simulation study suggests that the correspondence between the expected overall state and the observed sum score may be considerable. We simulated $N=500$ network structures of 10 nodes, with parameters for each network randomly drawn for each individual (see Appendix B for details). We then calculated the expected overall state for each person and simulated a single measurement occasion to obtain an observed sum score. Finally, we assessed the distributions of the expected overall states for each of the levels of the sum score. Results, as displayed in Fig. 4, show that in this case the ordering of the expected overall state by the sum score on the test resembles results obtained in the earlier IRT case.

Thus, some preliminary results, however modest and speculative, suggest that in complex networks, as in IRT, the sum score can be useful to assess the expected overall state of the network. Note that this is the case even though the individual networks nor the individual differences on the items should be expected to satisfy the axioms of the monotone homogeneity model. Further investigation is necessary to establish the precise psychometric properties of the sum score in network models. The simulation study suggests that in the assessment of psychological attributes, which are governed by a network structure rather than a latent variable, the sum score may play an important psychometric role.

### Sum Score in Retrospect

When it comes to the sheer ordering of persons, given a monotone unidimensional IRT model, one might very well use the sum score $X_{+}$ as the estimated latent variable $\hat{\theta }$, where the sum score seems to be a more robust ordinal estimator of the true ordering on $\theta$ than the WML estimate $\hat{\theta }$. For dichotomous item scores, the results are theoretically solid, and for polytomous item scores the results hold by approximation. In addition, the expected overall state of network constructs may also be assessed using sum scores. The fact that the sum score, by many psychometricians banned to the museum of psychometric history, is such a relevant alternative for the latent variable, if only for its simplicity and accessibility, raises the question why this ban could happen. Historical developments are difficult to explain, and speculation lurks around every corner. Could it be that the temptation of what is new blinds people for the merit of what exists and is available? We do not know for certain.

We are not saying that IRT models, other latent-variable models, or network models, must make way for the good old sum score, but rather that it would be a mistake to ban this simple performance measure in the presence of the proof of its usefulness. A simple explanation for the blind spot psychometrics seems to have for the merits of the sum score is that the papers discussing theoretical results are rather impenetrable due to their high math density, presumably clouding their results and what they mean. The simple fact is this. IRT provides the theoretical basis for the sum score that CTT does not give, CTT restricting itself to measurement by *fiat* (Torgerson, 1958). What CTT did right, however, is provide a solid theoretical framework for determining the degree to which test scores, in fact any scores, are influenced by random error. As we will see, this has not withheld part of psychometrics to be highly critical of the merits of CTT.

## The Sum Score and Reliability: A Relationship Made Confusing

We enter a field where the misunderstandings about what CTT is and what it accomplished are ubiquitous. We will discuss them at the end of this section. In addition, CTT seems to be incompletely or even incorrectly understood, a problem that stands apart from the misunderstandings but is of course serious and worrisome. Thus, we will start this section by defining CTT, followed by a discussion of the relation between CTT and the FA model, mainly because criticism on CTT and its reliability methods mainly come from that direction.

### Classical Test Theory Unchained and Revitalized

Since CTT has developed starting from the late 1800 s (Edgeworth, 1888), different definitions of true scores and error scores have been associated with it. This has created several versions of CTT, based on assumptions ranging from weak to highly restrictive. The restricted versions impose limitations on results derived from CTT, especially with respect to sum-score reliability. These restricted CTT versions have become the center of attention in the previous few decades, conveying the incorrect impression that CTT and CTT-based reliability are too restrictive to be of much practical use and must be replaced with alternative models and methods. One such nowadays popular alternative is the FA approach to reliability. Here, using the seminal book by Lord and Novick (1968, 1974) as the primary source, we take the opposite approach. Instead of presuming a latent-variable model in the background, we present a maximally unrestricted version of CTT and show how it can be used as a generic theory of measurement error that can be of use in the absence of the restrictions typically imposed by IRT and FA.

In several treatments of CTT, the true score is defined based on a model that explains how observable scores on items and tests are generated from population distributions. Lord and Novick (1968, 1974, chap. 2) discuss three such models but also notice that these score-generating models are not needed to formulate CTT and derive its results. We align with their conclusion and formulate a modern version of CTT with as few restrictions as possible, thus ignoring a score-generating model, that also includes the relation between reliability and the well-known coefficient $\alpha$ (Cronbach, 1951) as well as other lower-bound methods of approximating reliability. The important thing to note here is not the conclusion, but the fact that we are able to derive the relation in the absence of prior assumptions of the appropriateness or fit of a latent-variable model.

Our approach is based on Ellis (2021), who argues that multiple true score variables may exist for the same observable variable. We propose the following definitions, adapted from Ellis (2021, p. 873):

#### Definition 1

Consider a vector of random variables $\mathrm {\textbf{X}}=(X_{1},\cdots ,X_{J})$ with finite second moments. A set of random variables $\mathrm {\textbf{T}}=(T_{1},\cdots ,T_{J})$ with finite second moments is called a true score representation of $\mathrm {\textbf{X}}$ if the variable vector $\mathrm {\textbf{E}}=(E_{1},\cdots ,E_{J})$ defined by $\mathrm {\textbf{E}}=\mathrm {\textbf{X}}-\mathrm {\textbf{T}}$ satisfies covariance $\sigma \left( E_{j},T_{k} \right) =0$ for all $j,k=1,\cdots ,J$. The variables in $\mathrm {\textbf{X}}$ are called observable scores, the variables in $\mathrm {\textbf{T}}$ are called true scores, and the variables in $\mathrm {\textbf{E}}$ are called error scores. CTT is the study of relations between these variables.

Note that we do not commit to a particular specification of the nature of the observed, true, and error scores. For example, we do not assume that the true score is defined as a long run frequency, as in Lazarsfeld’s (1959) thought experiment in which a person is brainwashed in between test administrations to generate a long run frequency of test scores that underpins the person’s propensity distribution (Borsboom, Mellenbergh, & Van Heerden, 2004; Lord & Novick, 1968, 1974). While such an interpretation is consistent with the formal CTT assumptions, other interpretations that are consistent with the relevant relations between true and observed scores might be better defensible (see e.g., Rozeboom, 1966, and Van Bork et al., 2022, for some alternatives).

We will furthermore use the following definition:

#### Definition 2

We say that the errors are uncorrelated if $\sigma \left( E_{j},E_{k} \right) =0$ for all $j,k=\!1,\!\cdots \!,J;j\!\ne \! k.$

We continue using the definitions of the observable sum score and the true score of the sum score we introduced previously (i.e., $X_{+}=\sum \nolimits _{j=1}^J X_{j}$ and $T_{+}=\sum \nolimits _{j=1}^J T_{j} )$, and define the reliability of the observable sum score as the ratio of true and observed score variances, with variance denoted as $\sigma ^{2}$, so that,

$$\begin{aligned} &  \rho _{X_{+}}=\frac{\sigma ^{2}(T_{+})}{\sigma ^{2}(X_{+})}=1-\frac{\sigma ^{2}(E_{+})}{\sigma ^{2}(X_{+})}. \end{aligned}$$

Finally, as an example of a method that approximates the reliability, we define coefficient $\alpha$ as

$$\begin{aligned} &  \alpha = \frac{J}{J-1} \left( 1-\frac{\sum \nolimits _{j=1}^J {{\sigma ^{2}(X}_{j})} }{\sigma ^{2}(X_{+})} \right) . \end{aligned}$$

Importantly, reliability depends on the variance of true scores, which is unobservable. In contrast, coefficient $\alpha$ is a direct function of the observed variables. If $\alpha$ approximates $\rho _{X_{+}}$, the question is how they are related. One of the main results of CTT in this respect is the following theorem:

#### Theorem

For any true score representation with uncorrelated errors, $\alpha \le \rho _{X_{+}}$.

This is the well-known result stating that coefficient $\alpha$ is a lower bound to the reliability (Lord & Novick, 1968, 1974; they attribute this result to Guttman, 1945). *This is not a hypothesis, but a mathematical result that can be derived without any further assumptions*, as has been shown repeatedly (e.g., Novick & Lewis, 1967; Ten Berge & Sočan, 2004) and that we will reiterate later because it may not always have been understood completely. The theorem is correct given the Definitions 1 and 2, which must be considered mathematical tools needed to arrive at this and other CTT results. The interpretation of what the true score and the error scores might represent in the real world must be ignored. All that is needed to prove the theorem is to assume that the error scores do not correlate with the true scores and that they do not correlate with each other, but it is convenient to stay away from assigning meaning to these variables even though we call them true score and error to maintain the connection to CTT.

Mainly because the psychometric literature is so confusing about CTT, we explicate ten propositions that we do *not* assume here. We do not assume:

1. that the true score is equal to some expected value of an observable score; it might be, but it does not need to be;
2. replications of any kind;
3. the existence of parallel tests;
4. that the observable scores satisfy a 1-factor model or any other dimensional model;
5. that the observable scores reflect the same attribute, that they have anything in common, or that they have any meaning at all;
6. that the errors are independent; we assume merely that they are uncorrelated;
7. that the errors, the true scores, or the observable scores have a normal distribution; nor do we assume any other distribution for these variables;
8. that the errors have identical variances, that their variance is the same for every person, or that their variance is the same for two persons with the same true scores, nor do we assume any form of homoscedasticity; and
9. that the error scores are unpredictable; we merely assume that they cannot be predicted using linear regression from the true scores and other error scores of the test, but they might be predictable with nonlinear regression or from variables outside the test.
10. that the adjective “true” in “true score” implies that the theory correctly describes reality, that true scores coincide with psychological attributes, or that uncovering true scores is necessarily a target of psychological testing.

An example may illustrate our point. Suppose that some IRT model with latent-variable vector ${\varvec{\uptheta }}$ holds for $\mathrm {\textbf{X}}$, such that components of $\mathrm {\textbf{X}}$ are conditionally independent given ${\varvec{\uptheta }}.$ If we define $T_{j}=\mathbb {E} \left( X_{j} \vert {\varvec{\uptheta }}\right) ;j=1,\cdots ,J$, then $\mathrm {\textbf{T}}$ is a true score representation of $\mathrm {\textbf{X}}$ (see Definition 1) with uncorrelated errors(see Definition 2; that is, $\sigma \left( X_{j}-T_{j},X_{k}-T_{k} \right) =0$, for $j,k=1,\cdots ,J$; $j\ne k)$. Consequently, we may conclude that $\alpha \le \rho _{X_{+}}$ with these true score variables. Here, ${\varvec{\uptheta }}$ is not necessarily unidimensional; it may just as well be multidimensional. Even if ${\varvec{\uptheta }}$ is unidimensional, its relationship with the observable variables is not necessarily linear, as in a linear factor model; it may just as well be logistic or nonmonotone. The conclusion $\alpha \le \rho _{X_{+}}$ would be valid nonetheless in all these cases. See the next section for a more elaborate formulation of this.

From the above exposition, clearly CTT can be formulated as a measurement error theory and nothing else. It applies to any set of random variables for which one assumes that their observed values have a representation with underlying true scores $T_{j}$ such that the resulting measurement errors $E_{j}$ correlate 0 with the true scores $T_{k}$ ($j,k=1,\cdots ,J)$ and the other error scores $E_{k}$ ($j,k=1,\cdots ,J$; $k\ne j)$ in the representation. The representation of error scores thus captures an important property of how one would expect measurement error to behave. It is a remarkable feature of CTT that the theory manages to derive useful results from this set of assumptions. The results discussed thus far follow from the mathematics of the model and are independent of how one wishes to interpret the model, an observation valid for any measurement model. In addition, compared to IRT (and FA), CTT is a truly minimal model in terms of assumptions; for example, because it does not restrict dimensionality, the sum score of “blood pressure”, “driving speed”, “arithmetic test score”, and “anxiety score” is admissible in CTT, in the sense that relevant theorems, such as those concerning lower bounds, apply to this sum score as they would to any other score. Different scoring formats are allowed as well as different variable weighting. While many colleagues may object that such a weird test does not make sense—and they may be right from a *practical* point of view—the crucial insight is that CTT can operate in the absence of assumptions regarding the test’s dimensionality or factorial composition (Mellenbergh, 1994; Sijtsma & Pfadt, 2021).

### Classical Test Theory and Factor Analysis

Because it is reasonable to interpret CTT as a theory about measurement error, the most CTT can do is provide results concerning the influence of measurement error on sum scores. Thus, CTT is utilized to estimate the reliability of the sum score but also addresses topics depending on the reliability (and using some additional assumptions), such as the effect of lengthening (or shortening) the test on reliability, correcting the attenuation of correlations due to measurement error, and correcting for restriction of range in selection problems. Because CTT does not restrict the dimensionality of the measurement, psychologists and other researchers recognizing CTT’s limitations additionally use corrected item-total correlations (and the oblique multiple group method), principal component analysis, and FA (Sijtsma & Van der Ark, 2021). CTT’s reliability and the dimensionality methods such as FA became inseparable and gradually the idea developed that FA and CTT are two sides of the same coin. Indeed, given that CTT does not allow the assessment of dimensionality simply because this is not part of CTT and that, therefore, researchers need to use dimensionality assessment methods from outside of CTT, one could argue that there is a relation between CTT and FA, but we emphasize that this relation is not that one necessitates the other. Let us describe what this relation is, and what it is not:

1. CTT and FA have a common history in the work of such authoritative psychometricians as Spearman, Guttman and Cronbach, who often assumed a factor model when studying CTT. However, the fact that two models are studied by the same people for some decades does not make them the same model and it does not logically imply that they should always be used together.
2. Factor models usually assume uncorrelated errors, and therefore they imply a true score representation with uncorrelated errors. However, the converse is not true. In other words,

“FA $\Rightarrow$ CTT” is true, but “CTT $\Rightarrow$ FA” is false.

3. Furthermore, if the items are essentially $\tau$-equivalent ($\tau$ stands for true score), a mathematical equivalence condition on the items that is unrealistic for real data (and defined later), then the stronger relation $\alpha =\rho _{X_{+}}$holds, and essential $\tau$-equivalence implies that the item covariance matrix satisfies a 1-factor model with equal loadings. However, the inequality $\alpha \le \rho _{X_{+}}$ does not require any dimensional model and is useful even ignoring dimensionality altogether.
4. Similarly, for the Spearman-Brown prophecy formula, one would need parallel items, a mathematical equivalence condition even more restrictive than essential $\tau$-equivalence, which again implies a one-factor model. However, the Spearman-Brown formula is not necessarily used when one uses CTT because the test length is not necessarily changed. If the test length is changed, one can simply add or remove items until the desired reliability is obtained. Ellis and Sijtsma (2024) discuss conditions different from parallelism under which the Spearman-Brown formula functions reasonably well.
5. As we pointed out, users of CTT often use corrected item-total correlations, principal component analysis, or FA to assess whether the items relate strong enough with the other items to maintain them in the test. The use of these methods in combination with CTT by the same persons, does not entail a logical necessity, however. One might as well use nonlinear FA or IRT to study dimensionality, and still have a logically consistent analysis.

To summarize, CTT can be formulated as an error theory based on weak assumptions, which nevertheless allows for the derivation of interesting and even strong results. Methods such as FA but also IRT that, unlike CTT, address the dimensionality of an item set, can be used in addition to CTT but are not a mathematical part of CTT.

FA theorists interested in reliability estimation sometimes argue that the use of sum scores requires one to assume the highly restrictive 1-factor model so that the sum score is almost useless in the numerous practical test applications where this restrictive model is inconsistent with the data collected by means of the test. We already know from the previous section that this alleged implication is incorrect, but in this section, we take a closer look at the FA approach. A recent example comes from McNeish and Wolf (2020a; b), who claimed that the use of the sum score implies explicitly or implicitly assuming a 1-factor model with equal item loadings. Their parallel test model (ibid., p. 2290) translates to a 1-factor model assuming (1) that one latent variable, here a common true score, underlies all items, and (2) that the sum score can be written as the sum of *J* item scores weighted by equal item loadings, $a=1$, so that in FA notation we have

$$\begin{aligned} X_{+}=\sum \nolimits _{j=1}^J a X_{j}=\sum \nolimits _{j=1}^J X_{j} =\sum \nolimits _{j=1}^J {(T+E_{j})},\text { all } \sigma ^{2}(E_{j})=\sigma ^{2}. \end{aligned}$$

The restriction that all item-error variances are equal, $\sigma ^{2}(E_{j})=\sigma ^{2}$, however, is *not* an assumption that is necessary in CTT. A difference that is more important is that CTT is a nondimensional error model leaving entirely free which random variables to include in the sum score, each with their own true score. Again, we emphasize that we do not imply that tests be unintelligible blends of unrelated items. However, the point is that CTT allows this, and it is crucial in this discussion that we judge models by what they are, not by what people think or claim they are. Sijtsma and Van der Ark (2021, chap. 2) discuss the use of principle component analysis and FA (but IRT is also feasible) for assessing item set dimensionality, a practice familiar since decades in test construction.

Clearly, we disagree with the conclusion McNeish and Wolf (2020a; b) drew that using the sum score requires a restrictive 1-factor model with equal item loadings. Our conclusion is also evident from our discussion that a broad class of essentially unidimensional IRT models, much weaker than the parallel test model, justifies the use of the sum score. The parallel test model or the 1-factor model with equal loadings and equal item-error variances certainly implies that the sum score is usable. However, the opposite does not hold, because the 1-factor model merely defines one of the many sufficient conditions for sum score use but not a necessary condition. Another example of the broader support for use of the sum score comes from the network example discussed earlier in which the sum score is a useful summary of the expected network state, and no factor model was involved.

Interestingly, discussions of the sum score move quickly to discussions about which reliability method must be preferred (McNeish & Wolf, 2020a; b; Widaman & Revelle, 2022) and whether that reliability method must have its roots in CTT or in FA. This move is understandable in the context of an error model like CTT. Although reliability has been developed in the context of CTT and the sum score, there is no logical necessity to chain reliability to sum scores, simply because CTT is not limited to sum scores: Reliability and methods for estimating it from observable statistics are valid for any test score, which may include many other ways of forming a composite. Moreover, discussions about reliability divert attention from the issue whether the sum score is useful for assessment of psychological attributes. Next, we discuss the persistent problem of reliability lower bounds applied to the sum score.

### Reliability and Lower Bounds

In discussing the origin of lower-bound approximations to reliability, we skip much of the material reported in the literature but for more details, see Novick and Lewis (1967), Jackson and Agunwamba (1977), Ten Berge and Zegers (1978), Woodward and Bentler (1978), Bentler and Woodward (1980), Ten Berge and Sočan (2004), Sijtsma and Pfadt (2021), Sijtsma and Van der Ark (2021, chap. 2), and of course the seminal book by Lord and Novick (1968, 1974). We start by sharing our impression that in the critical literature on CTT reliability and coefficient $\alpha$ in particular, in addition to incompletely or incorrectly understanding CTT it is not always well understood where lower bounds such as coefficient $\alpha$ originate. A complicating factor in the critical discussions seems to be that it is not widely known that $\alpha$ is only one member of a large family of lower bounds, not even the best one in terms of its discrepancy defined as $\alpha -\rho _{X_{+}}$ (Sijtsma, 2009; Sijtsma & Pfadt, 2021). Such knowledge gaps may cause confusion leading discussions the wrong way.

We will sketch briefly what the assumptions underlying its derivation are, mainly following Novick and Lewis (1967). We derive an inequality, $L\le \rho _{X_{+}}$, where *L* is a generic notation that stands for lower bound and is a function of parameters that can be estimated from the data. The six reliability methods Guttman (1945) proposed under the names of $\lambda _{1},\cdots ,\lambda _{6}$, are all mathematical lower bounds (proofs in Jackson & Agunwamba, 1977; Sijtsma & Van der Ark, 2021, chap. 2), and more lower bounds based on CTT exist (see the references provided at the beginning of this subsection). Notation *L* can stand for any of them. Guttman’s $\lambda _{3}$ equals coefficient $\alpha$. Because $\alpha {\le \lambda }_{2}$, the latter method is closer to the reliability and, based on that knowledge alone, may be preferred to $\alpha$ (e.g., Sijtsma, 2009). The specific inequality we will derive will show that $L=\alpha$, so that we have shown that $\alpha$ is a lower bound. Some authors claim that the lower-bound theorem for $\alpha$ is false and that $\alpha$ can exceed the reliability. We note this is only possible when one departs from the CTT assumptions necessary to derive the lower bound. This triviality is true in all of mathematics: Abandoning the assumptions needed to arrive at a result will make it impossible to get there.

Recalling that the CTT model applies to any random variable, we focus on item scores and, as before, decompose them as $X_{j}=T_{j}+E_{j}$, in which $X_{j}$, $T_{j}$, and $E_{j}$ are random variables, and assume errors correlate 0 with true scores on all *J* items and errors on the other $J-1$ items except item *j*. Then, for sum score $X_{+}=\sum \nolimits _{j=1}^J X_{j}$, the corresponding sum of true scores equals $T_{+}=\sum \nolimits _{j=1}^J T_{j}$. We use the property of the variance of any variable that it is nonnegative. Hence, this is also true for the difference between two item true scores, so that $\sigma ^{2}(T_{j}-T_{k})\ge 0$. Because $\sigma ^{2}(T_{j}-T_{k})$ can be written as

$$\begin{aligned} \sigma ^{2}\left( T_{j}-T_{k} \right) =\sigma ^{2}(T_{j})+\sigma ^{2}(T_{k})-2\sigma (T_{j},T_{k}), \end{aligned}$$

the fact that the left-hand side must be nonnegative implies that,

$$\begin{aligned} \sigma ^{2}(T_{j})+\sigma ^{2}(T_{k})\ge 2\sigma (T_{j},T_{k}). \end{aligned}$$

Two remarks are important. First, this property is true for the difference of any pair of random variables, not just item scores. Second, so far, we have used item true scores depending on one item and have assumed nothing about the dimensionality of the whole set; that would require an item true score *T* independent of specific items. This remains the same throughout the derivation. Thus, the inequality holds irrespective of whether a unidimensional model is assumed or not. We refer the reader to details to be found in the sources mentioned, especially, Novick and Lewis (1967), but we will give a few steps of the longish derivation meanwhile urging the reader to flesh out the details for themselves. Then, the derivation involves the summation of both sides of the inequality across all item pairs, $j\ne k$, including both pairs (*j*, *k*) and (*k*, *j*) but excluding (*j*, *j*), all *j*, resulting in

1 $$\begin{aligned} \sum {\sum \nolimits _{j\ne k} {\left[ \sigma ^{2}(T_{j})+\sigma ^{2}(T_{k}) \right] \ge 2} \sum \sum \nolimits _{j\ne k} {\sigma (T_{j},T_{k})} }. \end{aligned}$$

We use two intermediate steps to rewrite the previous inequality. First, we take the sum of all combinations of $\left[ \sigma ^{2}(T_{j})+\sigma ^{2}(T_{k}) \right]$ including $j=k$, so that all variances appear 2*J* times; that is,

2 $$\begin{aligned} \sum \nolimits _{j=1}^J \sum \nolimits _{k=1}^J \left[ \sigma ^{2}(T_{j})+\sigma ^{2}(T_{k}) \right] =2J\sum \nolimits _{j=1}^J {\sigma ^{2}(T_{j})}. \end{aligned}$$

Second, we rewrite the left-hand side differently, splitting terms for which $j=k$ and terms for which $j\ne k$, so that,

3 $$\begin{aligned} \sum \nolimits _{j=1}^J \sum \nolimits _{k=1}^J \left[ \sigma ^{2}(T_{j})+\sigma ^{2}(T_{k}) \right] =2\sum \nolimits _{j=1}^J {\sigma ^{2}(T_{j})} +\sum \sum \nolimits _{j\ne k} \left[ \sigma ^{2}(T_{j})+\sigma ^{2}(T_{k}) \right] . \end{aligned}$$

Equating the right-hand sides of equations (2) and (3), we derive that,

4 $$\begin{aligned} \sum \sum \nolimits _{j\ne k} \left[ \sigma ^{2}(T_{j})+\sigma ^{2}(T_{k}) \right] =2(J-1)\sum \nolimits _{j=1}^J {\sigma ^{2}(T_{j})}. \end{aligned}$$

We replace the left-hand side of Equation (1) by the right-hand-side of Equation (4) and divide both sides of Equation (1) by $2(J-1)$, so that,

5 $$\begin{aligned} \sum \nolimits _{j=1}^J {\sigma ^{2}(T_{j})} \ge \frac{\sum \sum \nolimits _{j\ne k} {\sigma (T_{j},T_{k})} }{J-1}. \end{aligned}$$

Now, we use the well-known variance decomposition for linear combinations,

6 $$\begin{aligned} \sigma ^{2}\left( T_{+} \right) =\sum \nolimits _{j=1}^J {\sigma ^{2}(T_{j})} +\sum \sum \nolimits _{j\ne k} {\sigma (T_{j},T_{k})}. \end{aligned}$$

Substituting the right-hand side of Equation (5) for the first term on the right in Equation (6) yields,

7 $$\begin{aligned} \sigma ^{2}\left( T_{+} \right) \ge \frac{J}{J-1}\sum \sum \nolimits _{j\ne k} {\sigma (T_{j},T_{k})}. \end{aligned}$$

A well-known result in CTT is that $\sigma \left( T_{j},T_{k} \right) =\sigma (X_{j},X_{k})$, so here we introduce observable variables. Substituting for $\sigma (T_{j},T_{k})$ in Equation (7) and dividing both sides by the observable variance $\sigma ^{2}\left( X_{+} \right)$, yields the result *L* containing consisting of observables only, we hinted at before starting the derivation. The result is,

8 $$\begin{aligned} \frac{\sigma ^{2}\left( T_{+} \right) }{\sigma ^{2}\left( X_{+} \right) }\ge \frac{J}{J-1}\frac{\sum \sum \nolimits _{j\ne k} {\sigma (X_{j},X_{k})} }{\sigma ^{2}\left( X_+ \right) }. \end{aligned}$$

The reader will recognize reliability $\rho _{X_{+}}$ on the left-hand side and a function *L* that contains only observables on the right-hand side from which coefficient $\alpha$ can be recognized. This completes the derivation of the lower-bound theorem, which we note as

9 $$\begin{aligned} \alpha \le \rho _{X_{+}}. \end{aligned}$$

We consider the lower-bound theorems a beautiful result obtained in an error theory with few assumptions. An interesting question is when the inequality in the lower-bound theorem becomes an equality. This happens when we consider a boundary case of CTT in which all the items do share the same true score, a case CTT allows to happen but is not representative of CTT. This boundary case is known as essential $\tau$-equivalence (Lord & Novick, 1968, p. 50, Definition 2.13.8). This is a mathematical form of equivalence, defined as follows. Two items with scores $X_{j}$ and $X_{k}$ are essentially $\tau$-equivalent if, for every person *i* and some scalar $d_{jk}$,

$$\begin{aligned} T_{j}(i)=T_{k}(i)+d_{jk}. \end{aligned}$$

Essentially $\tau$-equivalent items do *not* necessarily have the same item-score variances (recall McNeish & Wolf’s assumption of equal variances), so that in general albeit not necessarily, $\sigma ^{2}(X_{j})\ne \sigma ^{2}(X_{k})$. It can be proven that for *J* items that are all essentially $\tau$-equivalent, we have that,

$$\begin{aligned} \alpha =\rho _{X_{+}}. \end{aligned}$$

We emphasize that this is a boundary case in CTT in which unidimensionality is satisfied. It does *not* specify a necessary assumption for interpreting and usefully employing coefficient $\alpha$ as a lower bound. Apparently, however, the fact that a unidimensional model is required for coefficient $\alpha$ to be a point estimate of reliability has given rise to the incorrect idea that, for lower bounds to reliability to be useful, the items in the test must satisfy essential $\tau$-equivalence. Next, some authors have equated this condition with a 1-factor model the items must satisfy or else the lower bound is useless (Cho, 2016; Cho and Kim, 2015; Dunn et al., 2014; Graham, 2006; Green and Yang, 2009; Miller, 1995). This conclusion is at least an exaggeration, because CTT is not a restricted 1-factor model (Hessen, 2023; Mellenbergh, 1994) but a much more general model that has proven highly useful for constructing tests and estimating the reliability of the test score used and applications thereof that are based on the reliability.

On a practical note, researchers often do not expect their item sets to be unidimensional but expect small deviations due to language and other skills needed to answer the items. In these cases, coefficient $\alpha$ or any other lower-bound methods are a little smaller than the CTT reliability, discrepancy $L-\rho _{X_{+}}$ depending on the degree to which the data are multidimensional. However, researchers will often do their best to assemble their test such that auxiliary skills and other influences have small effects on test performance, for example, by using language that is fully comprehensible to the complete population for which the test is meant. Because some degree of multidimensionality should always be expected, inferences based on the truth of the single factor model can be problematic, but lower-bound interpretations of coefficient $\alpha$ are always justified. This is precisely the situation in which lower bounds prove their usefulness. In addition, unless the test is very strongly multidimensional, lower bounds are not further off-target than a few hundredths at most (e.g., Sijtsma & Pfadt, 2021). Conservative reliability estimates do little damage (Widaman and Revelle, 2022): Knowing that the real reliability may be (a little) higher than the lower-bound value .87 hurts no-one but beware of overcorrection for attenuation of correlations if coefficient $\alpha$ (or another lower bound) is used.

### Lower Bounds in Retrospect

Some remarks seem to be in order. First, critics of lower bounds to the reliability, especially coefficient $\alpha$, apparently because it is the most popular method, seem to narrow CTT down to one of its boundary cases, which is the essential $\tau$-equivalence condition under which lower-bound methods theoretically equal reliability, $L=\rho _{X_{+}}$. This condition is then defined as a restricted 1-factor model and rejected because of its minimal chances of explaining real data well, and the conclusion is drawn that the lower bound, usually coefficient $\alpha$, is useless. What is ignored or perhaps not well understood, is that it is precisely in all the other, more realistic situations in which the data are inconsistent with an unrealistically restrictive 1-factor model, that reliability lower bounds prove their value by identifying an interval in which the true reliability lies: $\rho _{X_{+}}\in (L;1]$. If deviations from unidimensionality are *not too large*, then discrepancy $L-\rho _{X_{+}}$ is small, for lower bounds such as $\alpha$ no more than a few hundredths, but because such results are based on simulations, results may vary somewhat across different design choices (e.g., Malkewitz, Schwall, Meesters, & Hardt, 2023; Pfadt & Sijtsma, 2022). Sijtsma and Pfadt (2021) recommend using lower bounds when data are *approximately* unidimensional but notice that multidimensionality depends in complex ways on several item, test, and population features, making unambiguous conclusions difficult to attain. Because test constructors usually aim at measuring one attribute per test, we expect that especially in well-constructed tests unidimensionality is well approximated. This means that a lower-bound value of, say, .85, assures the researcher that reliability is anywhere between this value and 1. This is truly helpful information.

Second, criticism usually is targeted at coefficient $\alpha$, which can be understood because this is the most popular method of reliability estimation among a large array of lower-bound methods, and not the best method in terms of discrepancy (e.g., Sijtsma, 2009; Sijtsma & Pfadt, 2021). But what is difficult to understand is that critics have repeatedly claimed that, theoretically, $\alpha$ can be larger than $\rho _{X_{+}}$. Given the proof of the lower-bound theorem, $\alpha \le \rho _{X_{+}}$, such a claim is simply incorrect. Derivations of the opposite conclusion, which is that $\alpha >\rho _{X_{+}}$, can only be successful if one changes the assumptions under which the lower-bound theorem was derived previously. Of course, changing assumptions to define a model different from the CTT true-score-plus-random-error model is permissible if the math of the new model is correct, and reliability is redefined to adapt it to the variables of interest. For example, in FA one often replaces the CTT true score with the first common factor and then estimates the ratio of its variance to the variance of the sum score. In the presence of additional factors, such as correlated errors, this creates a situation in which one needs reliability estimation methods such as coefficient $\omega$ and not coefficient $\alpha$ or any of the other CTT lower bounds.

Third, it is seductive to interpret CTT as a measurement model. In particular, the infelicitous use of the adjective “true” in the true score component of the model suggests that the true score should coincide with a meaningful psychological attribute (Borsboom, 2005). However, since the critical assumptions of the model only relate to the behavior of errors, it is more useful to view CTT as a noise model rather than a measurement model. Interpreted this way, it is clear how CTT is useful to assess the reliability of sum scores, even in cases where one would not be inclined to interpret these as measurements in the usual sense of the term. This also broadens the applicability of CTT beyond the special case where a latent-variable model is indicated, for instance in the context of network constructs.

## Discussion and Take-Home Messages

The sum score is formally supported by a general class of essentially unidimensional IRT models, while other uses of sum scores, not founded in psychometric theory, are supported by their predictive power for job, school, and therapy performance, or stock exchange value (e.g., the Amsterdam Exchange Index) and scientific performance (e.g., the *h*-index, Hirsch, 2005). These other uses of sum scores do not employ a formal measurement model for their justification but rely solely on their practical usefulness, an approach originating decades ago (e.g., Cronbach & Gleser, 1957, 1965; Wiggins, 1973) when latent variables had a far less prominent role than today, and FA provided evidence for item set homogeneity followed by CTT reliability assessment. This is not to say that we must return to the days before latent-variable modeling became popular but rather that with the advent of a new approach the older approach does not automatically becomes obsolete, useless, or even reproachable. Criticisms of sum scores based on the idea that they are intuitive rather than scientific are premature and arguably incorrect. Even though we would rather wish that psychology and other disciplines that use tests and questionnaires had developed theories about attributes leading to well-founded measurement instruments, it is important to notice that researchers do their best to assemble item sets they believe to share the common core of the attribute of interest. They use psychometric methods such as corrected item-total correlations, principal component analysis, and FA to assess the homogeneity of their experimental item sets before estimating the sum score’s reliability and other psychometric properties. If an IRT model supports the resulting item set, the question is whether the latent variable can be used for the application envisaged. The stochastic ordering results concerning the sum score clarify that both the latent variable and the sum score can do about the same job when it comes to predicting behavior external to that producing the test results. Our preliminary results suggest that a similar case could be made based on network models.

We do not oppose alternative approaches to reliability determination and rather support any approach that aligns with the assumptions of the underlying psychometric model (e.g., Sijtsma & Pfadt, 2023). IRT is a convenient example. First, IRT allows estimating a standard error conditional on the latent variable depending on the suitability of the items at the latent variable location of interest. CTT also facilitates a sum score-dependent standard error but this knowledge, already dating back to the 1940 s and 1950 s has not become generally known (Mollenkopf, 1949; Thorndike, 1951; also, see Emons, 2023; Lek & Van der Schoot, 2018; Mellenbergh, 1996; Pfadt et al., 2023). We are not aware of a similar FA approach. FA and the broader structural equation modeling context focus on theory testing but not on test performance of individuals. This is more prominent in applications of CTT and IRT. Second, IRT models enable integrating different scales for the same attribute, such as in education where different school years made different exams having different items causing unequal difficulty levels. CTT using the sum score could also be used (Kolen and Brennan, 1995, 2004), but IRT is statistically better equipped.

Finally, our discussion of CTT, the sum score, and reliability lower bounds is not meant to downplay the value of alternative and more recent measurement models, but rather to provide an attempt to redirect attention to the value of older approaches that assume little and offer a lot, like CTT. We see two reasons why more recent measurement models provide an asset to the human sciences and psychology especially, and both reasons are highly persuasive. First, IRT offers the statistical tools that elegantly enable the equating of different scales for the same attribute and facilitate such impressive applications as adaptive testing, but also applications such as differential item function and person-fit research are easier using IRT in a way that CTT could not realize. Second, although still predominantly in the future, the substantive theory of the attribute one wishes to measure must dictate the psychometric model to be used for constructing the attribute’s scale (Sijtsma & Van der Ark, 2021). The many IRT models provide a wealth of possibilities, but different substantive theories might require approaches like networks and latent classes (Hagenaars & McCutcheon, 2002), with diagnostic classification models as interesting hybrids between latent class models and IRT.

The take-home messages of this article are the following:

- A broad class of IRT models formally justifies and supports the sum score, and thus supplies the foundation that CTT leaves open.
- The sum score may be useful in network models as well, even under conditions that do not correspond to the axioms of the monotone homogeneity model.
- Lower-bound methods for estimating reliability are derived with the minimum of assumptions and are perfectly in place in a theory-poor research area.
- Sum scores eventually derive their value from the degree to which they contribute to the prediction of societal relevant criterion events or behaviors.

## Figures and Tables

**Fig. 5:**
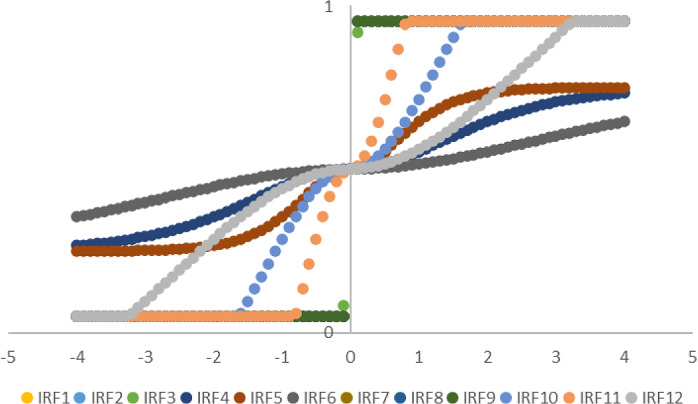
Examples of item response functions in the simulation study.

**Fig. 6:**
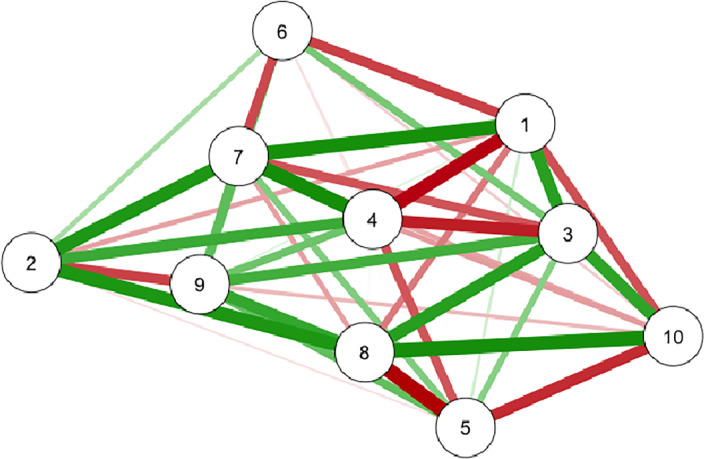
Small world network for one individual.

**Fig. 7:**
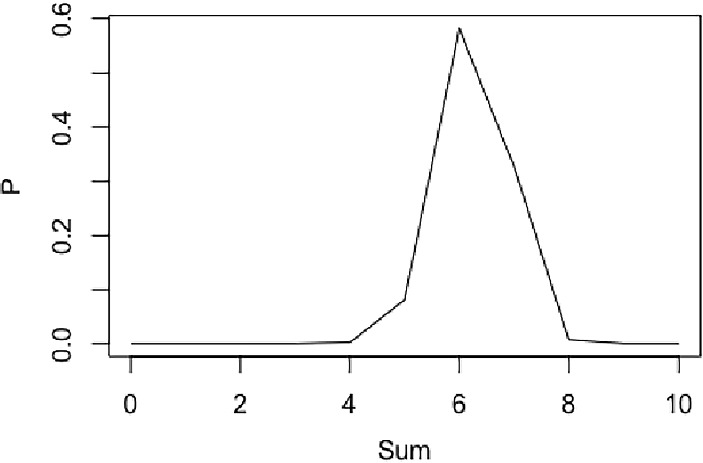
Full probability distribution over all sum scores.

**Fig. 8:**
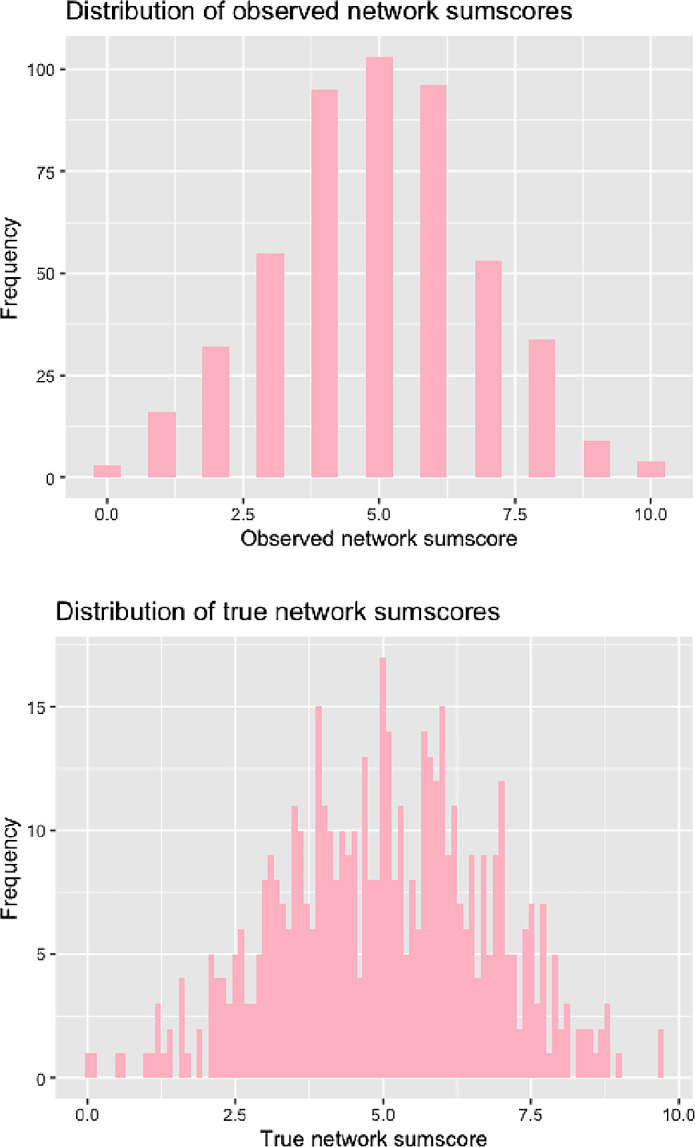
Univariate distributions for the observed (upper panel) and true (lower panel) network sum scores.

**Fig. 9:**
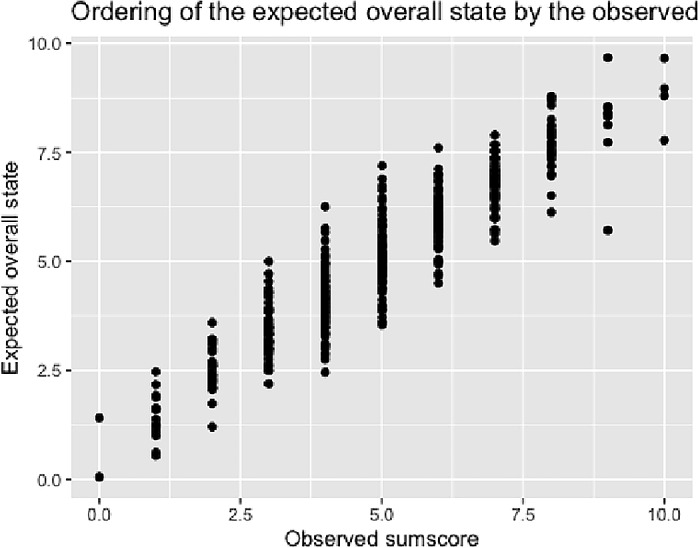
Expected overall state as function of observed sum score.

**Fig. 10:**
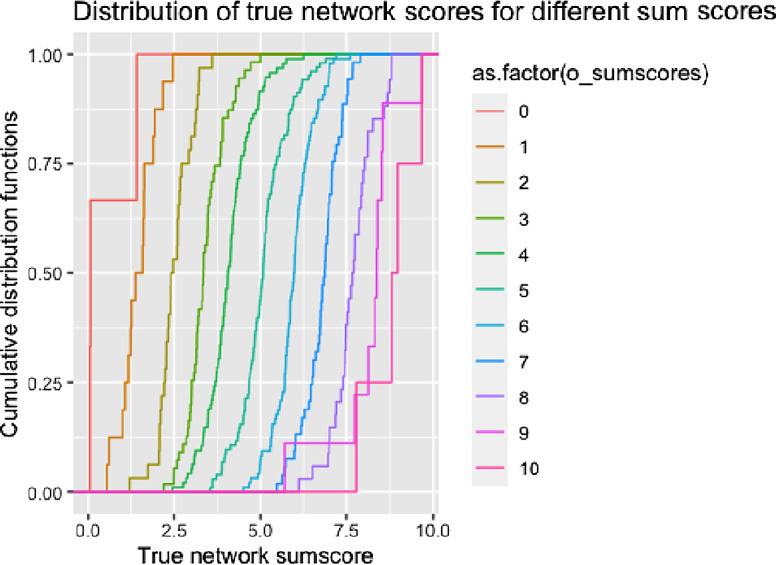
Cumulative distribution functions of the true network scores for different sum scores.

## Appendix A

In this appendix, we discuss a simulation study in which we compared the sum score $X_{+}$ with parametric estimates of latent variable $\theta$ in cases where the model is incorrectly specified, or the sample size is small. Table 1 shows the outcomes. The parametric estimation methods that we studied were weighted maximum likelihood (WML) and expected a posteriori (EAP); see column *est* of Table 1. The estimates were obtained with the R-package mirt (Chalmers, 2012), assuming a 2PL model. The data were generated with item response functions that may be specified incorrectly, which was controlled by two parameters named *pow* and *norm*. These parameters defined the item response functions as follows:

- If $pow=1$, then the data are generated with a 2PL model, which implies that the model is correctly specified in the estimation. In these cases, the model is

$$\begin{aligned} P\left( {X_{j}=1} \vert \theta \right) =\frac{\exp [\alpha _{j}\mathrm {\theta +}\beta _{j}\mathrm {]}}{1+\exp [\alpha _{j}\mathrm {\theta +}\beta _{j}\mathrm {]}}. \end{aligned}$$

- If $pow\ne 1$, then the data are generated using item response functions inconsistent with the 2PL model, so that the model is incorrectly specified in the estimation. In these cases, we defined the intermediary parameter
  $$\begin{aligned} &  p_{j}\left( \theta \right) =\frac{\exp [\alpha _{j}\mathrm {\theta +}\beta _{j}\mathrm {]}}{1+\exp [\alpha _{j}\mathrm {\theta +}\beta _{j}\mathrm {]}}, \end{aligned}$$

and the IRFs are now given by

$$\begin{aligned} P\left( {X_{j}=1} \vert \theta \right) =\min \left( 0.95,max\left( 0.05,\frac{sgn(p_{j}\left( \theta \right) -0.5)\left| p_{j}\left( \theta \right) -0.5 \right| ^{pow}}{norm} \right) \right) \end{aligned}$$

Figure 5 shows examples of possible item response functions. The item parameters were drawn from uniform distributions with $\alpha _{j}\sim Uniform(\alpha _{min},\alpha _{max})$, $\beta _{j}\sim Uniform(\beta _{min},\beta _{max})$, where $\alpha _{min},\alpha _{max}{, \beta }_{min},\beta _{max}$ are meta-parameters given in Table 1. The person parameters were drawn from a standard normal distribution. The number of items was $J=10$, $J=20$ or $J=40$. The number of persons was $N=100, N=500$ or$N=1000$.

Each row of Table 1 is defined by $N, J, \alpha _{min,}$ $\alpha _{max}, \beta _{min,} \beta _{max}, est, pow, norm$. For each row, we simulated 100 samples, and for each sample we computed the correlations $r_{1}=r(X_{+},\theta )$ and $r_{2}=r(\hat{\theta },\theta )$. Table 1 displays the means of $r_{1}$ and $r_{2}$, and the proportion of samples with $r_{1}>r_{2}$. We did not study all possible combinations of parameters but focused on WML with $N=500$ or $N=1000$ and $J=10$ or $J=40$. EAP presumes that the mean and variance of $\theta$ are known – which is often not true, and therefore we consider WML more interesting.

In all rows where the proportion of $r_{1}>r_{2}$ exceeds 0.50, the mean of $r_{1}$ also exceeds the mean of $r_{2}$, even though that may not be visible in the decimals displayed in Table 1. In these rows, the sum score may be viewed as superior to the parametric estimation method in column *est*. This happens in 78% of the rows, but note that we were searching for such cases, and we do not claim that this percentage is representative for all real situations. The point is that the sum score sometimes outperforms a parametric estimator, particularly if the model is incorrectly specified or if the sample size is small.

**Table 1:** Comparison of WLE or EAP with sum score $X_{+}$ as predictor of latent variable $\theta$ if item response functions are estimated.

Case	est	*N*	*J*	$\alpha _{min}$	$\alpha _{max}$	$\beta _{min}$	$\beta _{max}$	pow	norm	Mean $r_{1}$	Mean $r_{2}$	$r_{1}>r_{2}$
1	WLE	500	10	0.5	1.5	-1.5	1.5	1	1	0.792	0.788	0.65
2	EAP	500	10	0.5	1.5	-1.5	1.5	1	1	0.794	0.802	0.07
3	EAP	100	10	0.5	1.5	-1.5	1.5	1	1	0.795	0.783	0.66
4	WLE	1000	10	0.5	1.5	-1.5	1.5	1	1	0.796	0.796	0.53
5	WLE	500	20	0.5	1.5	-1.5	1.5	1	1	0.876	0.875	0.54
6	WLE	1000	20	0.5	1.5	-1.5	1.5	1	1	0.881	0.880	0.56
7	WLE	500	10	0.25	2	-2	2	1	1	0.783	0.792	0.21
11	WLE	500	10	0.25	2	-2	2	0.2	1	0.898	0.824	0.95
12	WLE	1000	10	0.25	2	-2	2	0.2	1	0.895	0.836	0.95
13	WLE	500	40	0.25	2	-2	2	0.2	1	0.962	0.894	1.00
14	WLE	1000	40	0.25	2	-2	2	0.2	1	0.963	0.896	1.00
15	WLE	500	10	0.25	2	-2	2	2	1	0.416	0.318	0.95
16	WLE	1000	10	0.25	2	-2	2	2	1	0.415	0.368	0.84
17	WLE	500	40	0.25	2	-2	2	2	1	0.684	0.677	0.57
18	WLE	1000	40	0.25	2	-2	2	2	1	0.682	0.692	0.20
21	WLE	500	10	0.25	2	-2	2	0.2	${0.5}^{0.2}$	0.898	0.822	0.96
22	WLE	1000	10	0.25	2	-2	2	0.2	${0.5}^{0.2}$	0.895	0.835	0.96
23	WLE	500	40	0.25	2	-2	2	0.2	${0.5}^{0.2}$	0.962	0.891	1.00
24	WLE	1000	40	0.25	2	-2	2	0.2	${0.5}^{0.2}$	0.963	0.897	1.00
25	WLE	500	10	0.25	2	-2	2	2	${0.5}^{0.2}$	0.822	0.818	0.52
26	WLE	1000	10	0.25	2	-2	2	2	${0.5}^{0.2}$	0.817	0.815	0.59
27	WLE	500	40	0.25	2	-2	2	2	${0.5}^{0.2}$	0.939	0.944	0.09
28	WLE	1000	40	0.25	2	-2	2	2	${0.5}^{0.2}$	0.939	0.944	0.05

## Appendix B

This simulation study, executed in R, was designed to analyze how informative the observed sum score is with respect to the expected sum score in a network context. In the simulation, each individual is characterized by a fully idiosyncratic network model. We parameterize this as an Ising model to obtain a probability distribution over all item-score patterns. Because different individuals have different networks, this distribution is different for each individual. The network model thus creates a probability distribution over the different possible sum scores. The *true network sum score* is defined as the expectation of this distribution. The observed item scores are conceptualized as a single realization of the item responses from a person’s probability distribution. The *observed network sum score* is the sum of these item scores. This setup allows us to assess how well differences in the observed sum score track differences in the expected sum score. The R markdown file to execute the simulation code is available at: http://bit.ly/3u94WdX. The next code shows how to create a small world network for one individual:

Figure 6 shows the resulting network.

For a single network, we can compute the expected sum score from the probability distribution specified by the Ising model using the function IsingSumLikelihood in the package IsingSampler. Here, and in the sequel, lines that start with ## contain the output of the preceding code:

Subsequently, we draw a single sample from this distribution, which we can think of as a single observation of the network:

In this sample, the person’s observed sum score equals 7.

Note that, at the level of the individual person, the true network sum score is a constant. The observed network sum score and the error scores are random variables over repeated draws from the distribution. As in CTT, we create the true network sum score to be a random variable by drawing individuals from a distribution. We do this by creating for each individual an entirely new network structure with new parameters. In the simulation reported in the main text, we simulated 500 individuals with different networks, and for each individual we recorded both the true network sum score and a single realized observed network sum score. Here, we (a) create a network for every individual in accordance with the code discussed earlier, (b) draw the edge weights for the present edges from a uniform distribution between 0 and 1, and (c) draw the thresholds from a normal distribution with a mean of $-2$ and a standard deviation of 10.

This simulation results in a joint distribution of the observed and true network sum scores. Figure 8 shows the univariate distributions for the observed and true network sum scores.

Now that we have a true network sum score as well as an observed sum score over the five items we assessed, we can investigate how they are related. First, we investigate whether the expectation of the true network sum scores increases in the observed network sum score. It turns out that this is the case here (Fig. 9).

To investigate stochastic ordering, we evaluate the cumulative distribution functions of the true network scores for different sum scores (Fig. 10). In this case, the observed sum score stochastically orders the true network sum score. Because we know the true network sum scores and the observed sum scores, we can define the network score reliability of the observed network score as the squared correlation between the observed and the true network sum scores. In this case, the network sum score has a reliability of. 87.

Note that this simulation is merely a proof of concept. In the current study, we have not evaluated model behavior across a wide range of conditions, nor did we provide a formal proof of stochastic ordering results.

## Declarations

### Data availability statement

The authors did not use empirical data for this article. The code used for simulating data and numerical examples is either available in the appendices or can be obtained on request from the first author or osf.io/tazws.

## References

[CR1] Bentler, P. M., & Woodward, J. A. (1980). Inequalities among lower bounds to reliability: With applications to test construction and factor analysis. *Psychometrika,**45*, 249–267.

[CR2] Binet, A., & Simon, Th. A. (1905). Méthodes nouvelles pour le diagnostic du niveau intellectuel des anormaux. *L’Année Psychologique,**11*, 191–244.

[CR3] Birnbaum, A. (1968). Some latent trait models and their use in inferring an examinee’s ability. In F. M. Lord, & M. R. Novick (1968). *Statistical theories of mental test scores* (pp. 396-479). Reading, MA: Addison-Wesley.

[CR4] Bollen, K. A. (1989). *Structural equations with latent variables*. New York, NY: Wiley.

[CR5] Bollen, K. A., & Pearl, J. (2013). Eight myths about causality and structural equation models. In S. L. Morgan (Ed.), *Handbook of causal analysis for social research* (pp. 301–328). Dordrecht, The Netherlands: Springer.

[CR6] Borsboom, D. (2005). *Measuring the mind. Conceptual issues in contemporary psychometrics*. Cambridge UK: Cambridge University Press.

[CR7] Borsboom, D., & Cramer, A. O. J. (2013). Network analysis: An integrative approach to the structure of psychopathology. *Annual Review of Clinical Psychology,**9*, 91–121. 10.1146/annurev-clinpsy-050212-185608 23537483

[CR8] Borsboom, D., Mellenbergh, G. J., & Van Heerden, J. (2004). The concept of validity. *Psychological Review,**111*, 1061–1071. 15482073 10.1037/0033-295X.111.4.1061

[CR9] Braun, H. I., & Mislevy, R. (2005). Intuitive test theory. *Phi, Delta, Kappan,**86*(7), 488–497. 10.1177/003172170508600705

[CR10] Brogden, H. E. (1946). Variation in test validity with variation in the distribution of item difficulties, number of items, and degree of their intercorrelation. *Psychometrika,**11*, 197–214. 20288942 10.1007/BF02290130

[CR11] Chalmers, R. P. (2012). mirt: A multidimensional item response theory package for the R environment. *Journal of Statistical Software,**48*(6), 1–29. 10.18637/jss.v048.i06

[CR12] Cho, E. (2016). Making reliability reliable: A systematic approach to reliability coefficients. *Organizational Research Methods,**19*, 651–682.

[CR13] Cho, E., & Kim, S. (2015). Cronbach’s coefficient alpha: Well known but poorly understood. *Organizational Research Methods,**18*, 207–230.

[CR14] Cronbach, L. J. (1951). Coefficient alpha and the internal structure of tests. *Psychometrika,**16*, 297–334.

[CR15] Cronbach, L. J., & Gleser, G. C. (1957, 1965). *Psychological tests and personnel decisions.* Urbana, IL: University of Illinois Press.

[CR16] Cronbach, L. J., & Warrington, W. G. (1952). Efficiency of multiple-choice tests as a function of spread of item difficulties. *Psychometrika,**17*, 127–147.

[CR17] Cramer, A. O. J., Van Borkulo, C. D., Giltay, E. J., Van Der Maas, H. L. J., Kendler, K. S., Scheffer, M., & Borsboom, D. (2016). Major depression as a complex dynamic system. *PLoS ONE*. 10.1371/journal.pone.0167490 27930698 10.1371/journal.pone.0167490 PMC5145163

[CR18] Dalege, J., Borsboom, D., van Harreveld, F., van den Berg, H., Conner, M., & van der Maas, H. L. J. (2016). Toward a formalized account of attitudes: The Causal Attitude Network (CAN) model. *Psychological Review,**123*, 2–22. 10.1037/a0039802 26479706 10.1037/a0039802

[CR19] diSessa, A. A. (1993). Toward an epistemology of physics. *Cognition and Instruction*, *10* (*2/3*), 105–225. https://www.jstor.org/stable/3233725

[CR20] Dunn, T. J., Baguley, T., & Brunsden, V. (2014). From alpha to omega: A practical solution to the pervasive problem of internal consistency estimation. *British Journal of Psychology,**105*, 399–412. 10.1111/bjop.12046 24844115 10.1111/bjop.12046

[CR21] Edgeworth, F. Y. (1888). The statistics of examinations. *Journal of the Royal Statistical Society,**51*, 599–635.

[CR22] Ellis, J. L. (2021). A test can have multiple reliabilities. *Psychometrika,**86*, 869–876. 10.1007/s11336-021-09800-2 34498211 10.1007/s11336-021-09800-2 PMC8636415

[CR23] Ellis, J. L., & Sijtsma, K. (2023). A test to distinguish monotone homogeneity from monotone multifactor models. *Psychometrika,**88*, 387–412. 10.1007/s11336-023-09905-w 36933110 10.1007/s11336-023-09905-w PMC10188426

[CR24] Ellis, J. L., & Sijtsma, K. (2024). Proof of reliability convergence to 1 at rate of Spearman-Brown formula for random test forms and irrespective of item pool dimensionality. *Psychometrika*. 10.1007/s11336-024-09956-7 PMC11458731 38472632

[CR25] Emons, W. H. M. (2023). Methods for estimating conditional standard errors of measurement and some critical reflections. In L. A. van der Ark, W. H. M. Emons, & R. R. Meijer (Eds.), *Essays on contemporary psychometrics* (pp. 195–216). New York, NY: Springer.

[CR26] Ferguson, G. A. (1942). Item selection by the constant process. *Psychometrika,**7*, 19–29.

[CR27] Finnemann, A., Borsboom, D., Epskamp, S., & van der Maas, H. L. J. (2021). The theoretical and statistical Ising model: A practical guide in R. *Psych,**3*, 593–617. 10.3390/psych3040039

[CR28] Finney, D. J. (1944). The application of probit analysis to the results of mental tests. *Psychometrika,**9*, 31–39.

[CR29] Fischer, G. H. (1974). *Einführung in die Theorie psychologischer Tests (Introduction to the theory of psychological tests)*. Bern, Switserland: Huber.

[CR30] Fischer, G. H. (1995). Derivations of the Rasch model. In G. H. Fischer & Molenaar, I. W. (Eds.) (1995). *Rasch models. Foundations, recent developments, and applications* (pp. 15–38). New York: Springer-Verlag.

[CR31] Graham, J. M. (2006). Congeneric and (essentially) tau-equivalent estimates of score reliability. What they are and how to use them. *Educational and Psychological Measurement,**66*, 930–944. 10.1177/0013164406288165

[CR32] Grayson, D. A. (1988). Two-group classification in latent trait theory: Scores with monotone likelihood ratio. *Psychometrika,**53*, 383–392.

[CR33] Green, S. B., & Yang, Y. (2009). Reliability of summed item scores using structural equation modeling: An alternative to coefficient alpha. *Psychometrika,**74*, 155–167. 10.1007/S11336-008-9099-3

[CR34] Guttman, L. (1945). A basis for analyzing test–retest reliability. *Psychometrika,**10*, 255–282. 21007983 10.1007/BF02288892

[CR35] Hagenaars, J. A., & McCutcheon, A. L. (Eds.). (2002). *Applied latent class analysis*. Cambridge, UK: Cambridge University Press.

[CR36] Hemker, B. T. (2023). To a or not to a: On the use of the total score. In L. A. van der Ark, W. H. M. Emons, & R. R. Meijer (Eds.), *Essays on contemporary psychometrics* (pp. 251–270). New York, NY: Springer.

[CR37] Hemker, B. T., Sijtsma, K., Molenaar, I. W., & Junker, B. W. (1996). Polytomous IRT models and monotone likelihood ratio of the total score. *Psychometrika,**61*, 679–693.

[CR38] Hemker, B. T., Sijtsma, K., Molenaar, I. W., & Junker, B. W. (1997). Stochastic ordering using the latent trait and the sum score in polytomous IRT models. *Psychometrika,**62*, 331–347.

[CR39] Hessen, D. J. (2023). A new expression and interpretation of coefficient omega under the congeneric one-factor model. In L. A. van der Ark, W. H. M. Emons, & R. R. Meijer (Eds.), *Essays on contemporary psychometrics* (pp. 111–118). New York, NY: Springer.

[CR40] Hirsch, J. E. (2005). An index to quantify an individual’s scientific research output. *Proceedings of the National Academy of Sciences of the United States of America,**102*, 16569–16572. 10.1073/pnas.0507655102 16275915 10.1073/pnas.0507655102 PMC1283832

[CR41] Holland, P. W., & Hoskens, M. (2003). Classical test theory as a first-order item response theory: Application to true-score prediction from a possibly nonparallel test. *Psychometrika,**68*, 123–149.

[CR42] Holland, P. W., & Rosenbaum, P. R. (1986). Conditional association and unidimensionality in monotone latent variable models. *The Annals of Statistics,**14*, 1523–1543.

[CR43] Huynh, H. (1994). A new proof for monotone likelihood ratio for the sum of independent Bernoulli random variables. *Psychometrika,**59*, 77–79.

[CR44] Ising, E. (1925). Beitrag zur Theorie des Ferromagnetismus. *Zeitschrift f*ü*r Physik, 31*, 253–258.

[CR45] Jackson, P. H., & Agunwamba, C. C. (1977). Lower bounds for the reliability of the total score on a test composed of non-homogeneous items: I: Algebraic lower bounds. *Psychometrika,**42*, 567–578.

[CR46] Junker, B. W. (1991). Essential independence and likelihood-based ability estimation for polytomous items. *Psychometrika,**56*, 255–278.

[CR47] Junker, B. W. (1993). Conditional association, essential independence and monotone unidimensional item response models. *The Annals of Statistics,**21*, 1359–1378.

[CR48] Kolen, M. J., & Brennan, R. L. (1995). *Test equating. Methods and practices*. New York, NY: Springer.

[CR49] Kolen, M. J., & Brennan, R. L. (2004). *Test equating, scaling, and linking. Methods and practices.* New York, NY: Springer.

[CR50] Lawley, D. N. (1943). On problems connected with item selection and test construction. *Proceedings of the Royal Society of Edinburgh,**61*, 73–287.

[CR51] Lazarsfeld, P. F. (1959). Latent structure analysis. In S. Koch (Ed.), *Psychology: A study of a science. * (Vol. 3). New York, NY: McGraw-Hill.

[CR52] Lek, K. M., & Van de Schoot, R. (2018). A comparison of the single, conditional and person-specific standard error of measurement: What do they measure and when to use them? *Frontiers in Applied Mathematics and Statistics,**4*, 40. 10.3389/fams.2018.00040

[CR53] Ligtvoet, R. (2022). Incomplete tests of conditional association for the assessment of model assumptions. *Psychometrika,**87*, 1214–1237. 10.1007/s11336-022-09841-1 35124767 10.1007/s11336-022-09841-1 PMC9636116

[CR54] Ligtvoet, R. (2022b). The sum scores and discretization of variables under the linear normal one-factor model. In M. Wiberg, D. Molenaar, J. González, J.-S. Kim, & H. Hwang (Eds), *Quantitative psychology. The 86th Annual Meeting of the Psychometric Society, Virtual, 2021* (pp. 227–235). Springer. 10.1007/978-3-031-04572-1_17

[CR55] Lord, F. M. (1952). A theory of test scores. *Psychometric Monograph No. 7,* Psychometric Society.

[CR56] Lord, F. M. (1980). *Applications of item response theory to practical testing problems*. Hillsdale, NJ: Erlbaum.

[CR57] Lord, F.M., & Novick, M. R. (1968, 1974). *Statistical theories of mental test scores*. Reading, MA: Addison-Wesley.

[CR58] Malkewitz, C. P., Schwall, P., Meesters, C., & Hardt, J. (2023). Estimating reliability: Estimating Cronbach’s , McDonald’s and the greatest lower bound. *Social Sciences & Humanities Open*. 10.1016/j.ssaho.2022.100368

[CR59] Marsman, M., Borsboom, D., Kruis, J., Epskamp, S., van Bork, R., Waldorp, L. J., van der Maas, H. L. J., & Maris, G. (2018). An introduction to network psychometrics: Relating Ising network models to item response theory models. *Multivariate Behavioral Research,**53*, 15–35. 29111774 10.1080/00273171.2017.1379379

[CR60] Marsman, M., & Rhemtulla, M. (2022). Guest Editors’ Introduction to The Special Issue “Network Psychometrics in Action”: Methodological Innovations Inspired by Empirical Problems. *Psychometrika*, *87*(1), 1–11. 10.1007/s11336-022-09861-x 10.1007/s11336-022-09861-x PMC9021145 35397084

[CR61] McNeish, D. (2023). Psychometric properties of sum scores and factor scores differ even when their correlation is 0.98: A response to Widaman and Revelle. *Behavior Research Methods,**55*, 4269–4290. 10.3758/s13428-022-02016-x 36394821 10.3758/s13428-022-02016-x

[CR62] McNeish, D., & Wolf, M. G. (2020a). Thinking twice about sum scores. *Behavior Research Methods,**52*, 2287–2305. 10.3758/s13428-020-01398-0 32323277 10.3758/s13428-020-01398-0

[CR63] McNeish, D., & Wolf, M. G. (2020b). Corrigendum to: Thinking twice about sum scores. *Behavior Research Methods,**52*, 2674. 10.3758/s13428-020-01468-3 32869138 10.3758/s13428-020-01468-3

[CR64] Mellenbergh, G. J. (1994). A unidimensional latent trait model for continuous item responses. *Multivariate Behavioral Research,**29*, 223–236. 10.1207/s15327906mbr2903_2 26765136 10.1207/s15327906mbr2903_2

[CR65] Mellenbergh, G. J. (1996). Measurement precision in test score and item response models. *Psychological Methods,**1*, 293–299. 10.1037/1082-989X.9.1.116 15053722

[CR66] Miller, M. B. (1995). Coefficient alpha: A basic introduction from the perspectives of classical test theory and structural equation modeling. *Structural Equation Modeling,**2*, 255–273. 10.1080/10705519509540013

[CR67] Mollenkopf, W. G. (1949). Variation on the standard error of measurement. *Psychometrika,**14*, 189–229. 24536852 10.1007/BF02289153

[CR68] Novick, M. R., & Lewis, C. (1967). Coefficient alpha and the reliability of composite measurements. *Psychometrika,**32*, 1–13. 5232569 10.1007/BF02289400

[CR69] Pfadt, J. M., Molenaar, D., Hurks, P., & Sijtsma, K. (2023). A tutorial on the precision of the measurement of individuals using test scores (manuscript in preparation).

[CR70] Pfadt, J. M., & Sijtsma, K. (2022). Statistical properties of lower bounds and factor analysis methods for reliability estimation. In M. Wiberg, D. Molenaar, J. González, J.-S. Kim, & H. Hwang (Eds.), *Quantitative psychology: The 86**Annual Meeting of the Psychometric Society, virtual 2021* (pp. 51–63). New York, NY: Springer.

[CR71] Rasch, G. (1960). *Probabilistic models for some intelligence and attainment tests*. Copenhagen: Nielsen & Lydiche.

[CR72] Rasch, G. (1968). An individualistic approach to item analysis. In P. F. Lazarsfeld & N. W. Henry (Eds.), *Latent structure analysis* (pp. 89–107). Boston, MA: Houghton Mifflin.

[CR73] Richardson, M. W. (1936). The relation between the difficulty and the differential validity of a test. *Psychometrika,**1*, 33–49.

[CR74] Rozeboom, W. W. (1966). Scaling theory and the nature of measurement. *Synthese,**16*, 170–233.

[CR75] Sijtsma, K. (2009). On the use, the misuse, and the very limited usefulness of Cronbach’s alpha. *Psychometrika,**74*, 107–120. 20037639 10.1007/s11336-008-9101-0 PMC2792363

[CR76] Sijtsma, K., & Molenaar, I. W. (2002). *Introduction to nonparametric item response theory*. Thousand Oaks, CA: Sage.

[CR77] Sijtsma, K., & Pfadt, J. M. (2021). Part II: On the use, the misuse, and the very limited usefulness of Cronbach’s alpha: Discussing lower bounds and correlated errors. *Psychometrika,**86*, 843–860. 10.1007/s11336-021-09789-8 34387809 10.1007/s11336-021-09789-8 PMC8636457

[CR78] Sijtsma, K., & Pfadt, J. M. (2023). Reliability. In R. Tierney, F. Rizvi, & K. Ercikan (Eds.), *International**encyclopedia of education* (4 edition), *Quantitative Research and Educational Measurement* (pp. 657-669). Amsterdam, the Netherlands: Elsevier. 10.1016/B978-0-12-818630-5.10004-1

[CR79] Sijtsma, K., & Van der Ark, L. A. (2021). *Measurement models for psychological attributes*. Boca Raton, FL: Chapman & Hall/CRC.

[CR80] Stout, W. F. (1990). A new item response theory modeling approach with applications to unidimensionality assessment and ability estimation. *Psychometrika,**55*, 293–325.

[CR81] Stout, W. F. (2002). Psychometrics: From practice to theory and back. *Psychometrika,**67*, 485–518.

[CR82] Ten Berge, J. M. F., & Sočan, G. (2004). The greatest lower bound to the reliability of a test and the hypothesis of unidimensionality. *Psychometrika,**69*, 613–625.

[CR83] Ten Berge, J. M. F., & Zegers, F. E. (1978). A series of lower bounds to the reliability of a test. *Psychometrika,**43*, 575–579.

[CR84] Thorndike, R. L. (1951). Reliability. In E. F. Lindquist (Ed.), *Educational measurement* (pp. 560–620). Washington DC: American Council on Education.

[CR85] Torgerson, W. S. (1958). *Theory and methods of scaling*. New York, NY: Wiley.

[CR86] Unlü, A. (2008). A note on monotone likelihood ratio of the total score variable in unidimensional item response theory. *British Journal of Mathematical and Statistical Psychology,**61*, 179–187. 10.1348/000711007X173391 17535477 10.1348/000711007X173391

[CR87] Van Bork, R., Rhemtulla, M., Sijtsma, K., & Borsboom, D. (2022). A causal theory of error scores. *Psychological Methods*. 10.1037/met0000521 35878074 10.1037/met0000521

[CR88] Van der Ark, L. A. (2005). Practical consequences of stochastic ordering of the latent trait under various polytomous IRT models. *Psychometrika,**70*, 283–304.

[CR89] Van der Ark, L. A., & Bergsma, W. P. (2010). A note on stochastic ordering of the latent trait using the sum of polytomous item scores. *Psychometrika,**75*, 272–279.

[CR90] Van der Linden, W. J. (Ed.) (2016). *Handbook of item response theory: Volume One. Models*. Boca Raton, FL: Chapman & Hall/CRC.

[CR91] Van der Linden, W. J., & Hambleton, R. K. (Eds.). (1997). *Handbook of modern item response theory*. New York, NY: Springer.

[CR92] Van der Maas, H. L. J., Dolan, C. V., Grasman, R. P. P. P., Wicherts, J. M., Huizenga, H. M., & Raijmakers, M. E. J. (2006). A dynamical model of general intelligence: The positive manifold of intelligence by mutualism. *Psychological Review,**113*, 842–861. 17014305 10.1037/0033-295X.113.4.842

[CR93] Van der Maas, H. L. J., Kan, K.-J., & Borsboom, D. (2014). Intelligence is what the intelligence test measures. Seriously. *Journal of Intelligence,**2*, 12–15.

[CR94] Warm, T. A. (1989). Weighted likelihood estimation of ability in item response models. *Psychometrika,**54*, 427–450.

[CR95] Widaman, K. F., & Revelle, W. (2022). Thinking thrice about sum scores, and then some more about measurement and analysis. *Behavior Research Methods*. 10.3758/s13428-022-01849-w 35469086 10.3758/s13428-022-01849-w PMC10027776

[CR96] Widaman, K. F., & Revelle, W. (2023). Thinking about sum scores yet again, maybe the last time, we don’t know, oh no . . .: A comment on McNeish (2023). *Educational and Psychological**Measurement*. 10.1177/00131644231205310 10.1177/00131644231205310 PMC11268387 39055096

[CR97] Wiggins, J. S. (1973). *Personality and prediction: Principles of personality assessment*. Reading, MA: Addison-Wesley.

[CR98] Woodward, J. A., & Bentler, P. M. (1978). A statistical lower-bound to population reliability. *Psychological Bulletin,**85*, 1323–1326. 734016

